# Energy Harvesting Technologies for Structural Health Monitoring of Airplane Components—A Review

**DOI:** 10.3390/s20226685

**Published:** 2020-11-22

**Authors:** Saša Zelenika, Zdenek Hadas, Sebastian Bader, Thomas Becker, Petar Gljušćić, Jiri Hlinka, Ludek Janak, Ervin Kamenar, Filip Ksica, Theodora Kyratsi, Loucas Louca, Miroslav Mrlik, Adnan Osmanović, Vikram Pakrashi, Ondrej Rubes, Oldřich Ševeček, José P.B. Silva, Pavel Tofel, Bojan Trkulja, Runar Unnthorsson, Jasmin Velagić, Željko Vrcan

**Affiliations:** 1 University of Rijeka, Faculty of Engineering, Vukovarska 58, 51000 Rijeka, Croatia; pgljuscic@riteh.hr (P.G.); ekamenar@riteh.hr (E.K.); zeljko.vrcan@riteh.hr (Ž.V.); 2 University of Rijeka, Centre for Micro- and Nanosciences and Technologies, Radmile Matejčić 2, 51000 Rijeka, Croatia; 3 Faculty of Mechanical Engineering, Brno University of Technology, Technická 2896/2, 61669 Brno, Czech Republic; hlinka@fme.vutbr.cz (J.H.); janak@fme.vutbr.cz (L.J.); Filip.Ksica@vutbr.cz (F.K.); ondrej.rubes@vut.cz (O.R.); sevecek@fme.vutbr.cz (O.Š.); 4 Department of Electronics Design, Mid Sweden University, Holmgatan 10, 85170 Sundsvall, Sweden; Sebastian.Bader@miun.se; 5 Thobecore Consulting & Research, 27711 Osterholz-Scharmbeck, Germany; dr-thomas-becker@web.de; 6 Department of Mechanical and Manufacturing Engineering, University of Cyprus, 1 Panepistimiou Ave, 2109 Nicosia, Cyprus; kyratsi@ucy.ac.cy (T.K.); lslouca@ucy.ac.cy (L.L.); 7 Centre of Polymer Systems, Tomas Bata University in Zlín, 76001 Zlín, Czech Republic; mrlik@utb.cz; 8 Faculty of Electrical Engineering, University of Sarajevo, Zmaja od Bosne bb, 71000 Sarajevo, Bosnia and Herzegovina; adnan.osmanovic@etf.unsa.ba (A.O.); jasmin.velagic@etf.unsa.ba (J.V.); 9 Dynamical Systems and Risk Laboratory, School of Mechanical and Materials Engineering, Engineering Building Belfield, University College Dublin, Dublin 4, Ireland; vikram.pakrashi@ucd.ie; 10 Centre of Physics of University of Minho and Porto (CF-UM-UP), Campus de Gualtar, 4710-057 Braga, Portugal; josesilva@fisica.uminho.pt; 11 Faculty of Electrical Engineering and Communications, Brno University of Technology, Technická 3058/10, 61600 Brno, Czech Republic; tofel@feec.vutbr.cz; 12 Faculty of Electrical Engineering and Computing, University of Zagreb, Unska 3, 10000 Zagreb, Croatia; bojan.trkulja@fer.hr; 13 Faculty of Industrial Engineering, Mechanical Engineering and Computer Science, School of Engineering and Natural Sciences, University of Iceland, Saemundargotu 2, 102 Reykjavik, Iceland; runson@hi.is

**Keywords:** energy harvesting, airplane, non-destructive evaluation, kinetic, thermoelectric, solar, smart skin, power management

## Abstract

With the aim of increasing the efficiency of maintenance and fuel usage in airplanes, structural health monitoring (SHM) of critical composite structures is increasingly expected and required. The optimized usage of this concept is subject of intensive work in the framework of the EU COST Action CA18203 “Optimising Design for Inspection” (ODIN). In this context, a thorough review of a broad range of energy harvesting (EH) technologies to be potentially used as power sources for the acoustic emission and guided wave propagation sensors of the considered SHM systems, as well as for the respective data elaboration and wireless communication modules, is provided in this work. EH devices based on the usage of kinetic energy, thermal gradients, solar radiation, airflow, and other viable energy sources, proposed so far in the literature, are thus described with a critical review of the respective specific power levels, of their potential placement on airplanes, as well as the consequently necessary power management architectures. The guidelines provided for the selection of the most appropriate EH and power management technologies create the preconditions to develop a new class of autonomous sensor nodes for the in-process, non-destructive SHM of airplane components.

## 1. Introduction

The aeronautic industry increasingly relies on composite materials (with the current rates at even 50% of the overall structures’ weight) that result in weight reduction but also imply complex damage mechanics. This induces an increased use of in-process structural health monitoring (SHM) systems aimed at non-destructive testing (NDT) of the state of airplane components [1,2]. NDT is defined in this frame as “the examination of an object with technology that does not affect the object’s future usefulness” (American Society of Non-destructive Testing), whereas SHM is defined as “the process of acquiring and analysing data from on-board sensors to evaluate the health of a structure” (Committee on Structural Health Monitoring and Management) [3]. NDT is usually carried out during shutdowns or maintenance procedures, when the monitored structure is not operating but it is subjected, in a controlled manner and for relatively short periods of time, to conditions similar to those during operation, while the used sensors are often attached temporarily to the structure. SHM, in turn, utilizes on-board sensor systems permanently attached to the monitored structure, taking measurements during operation and generating data automatically.

Since SHM takes place during operation, the system, requiring multidisciplinary knowledge, has to be integrable onto or into the structure with included signal processing and data management components, with the hence transmitted information strongly dependent on its purpose and importance for in-flight conditions. Currently, mainly due to the fact that most of the research and development takes place in laboratory conditions on simplified structures, there is still a marked gap between the advance of the knowhow on one hand and the industrial applications of SHM technologies on the other. The requirements for a localized damage detection in large-scale structures induces, concurrently, a considerable growth of the sensor array size. All of these factors affect the choice of suitable techniques, sensor types and signal processing procedures so as to attain functional SHM systems usable in real-world applications [4].

SHM procedures can encompass various technologies, each with its own limitations in terms of hardware and software requirements, overall applicability and cost effectiveness in civil aviation industry. In aerospace industry, the most promising SHM technologies, each utilizing different types of sensors and used for a different kind of monitoring (local or global monitoring, impact detection), are those based on wave propagation (including acoustic emission and ultrasonics), optical fibres, electro-mechanical impedance, electric resistance, coating monitoring, vacuum monitoring, and eddy current-based systems [3,4,5,6,7].

As especially promising for airplane industry, wave propagation SHM techniques, whose needed power levels, depending on the complexity of the considered SHM system, have been estimated to be in the range between a few and a couple of hundred milliwatts (mW) [8], are subject of intensive work in the framework of the EU COST Action CA18203 “Optimising Design for Inspection” (ODIN) [9]. The wave propagation techniques can be based on measuring signals, generated by events occurring within the structure itself (acoustic emission) or generated externally by transducers in proximity of the sensor (guided wave propagation) that are propagated through the structure. Acoustic emission (AE) stands out over other techniques due to its ability to detect dynamic changes within the structure associated with the damage, the ability to detect and localize the sources of damage, as well as the ability to cover large areas with a relatively low number of sensors. On the other hand, while AE can provide information about damage being developed in the structure, and it has thus been successfully used in civil engineering, wind turbines, and in aerospace developments [10,11,12], it cannot generally do so for the damage currently present in the structure. The guided wave propagation technique relies, in turn, on the usage of an external source of ultrasonic elastic waves that propagate through the structure, and it can be used to detect the damage already present within the structure, such as the airplane wing [13]. Piezoelectric materials are commonly used in this frame as both signal generating elements as well as sensors [14,15]. Guided waves are widely used for on-site or laboratory testing and the inspection of composite structures [16,17], as well as for the detection of crack initiation and growth in metallic structures of airplanes [18]. However, this method usually requires, a specific arrangement of the sensor array [19] and there are significant challenges in terms of the respective signal processing and extraction. In fact, the detection of real-world degradation initiated by microscopic defects requires the extraction of complex nonlinear features of the waves, hence increasing significantly the processing time [20].

Viable alternative SHM techniques, whose potential usage in the monitoring of aeronautic components is being studied, include optical fibres-based systems [21,22,23,24,25,26], systems founded on the measurement of impedance, based on piezoelectric patches attached to the structure acting, again, simultaneously as actuators and sensors [27,28], systems based on the direct measurement of structural vibrations and the respective modal parameters of the studied structure, which is especially common in research concerning autonomous sensor nodes using the wireless transmission of data [29], and smart skin systems based on lightweight and highly stretchable piezoelectric sensor networks used for high accuracy monitoring of impacts on the aircraft [30,31].

The addressed SHM sensors then have to be coupled to suitable data elaboration (signal processing and data logging) modules, whose power requirements can be estimated to be in the range from a few mW [32] to, depending on the foreseen requirements, a couple of hundred mW, as well as (increasingly wireless) communication systems. In fact, the usage of wireless in-process SHM systems could have a huge impact on the reduction of the weight of wiring (currently an A380 airplane has 530 km of wiring) and thus on the efficiency of fuel consumption [9]. This approach would also allow enhancing system safety by means of dissimilar redundancy (wired and wireless), as well as by providing the possibility to wirelessly access some sensors that cannot be otherwise connected (e.g., on moving parts). The adoption of such a methodology contributes concurrently to increasing the safety and reliability of airplanes by reducing, via predictive maintenance—a crucial process in determining the probability of failure while providing recommendations on the necessary maintenance frequency with respect to the state of the structure—, the operational costs [8].

From the technological point of view, two wireless communication options can be distinguished: the wireless avionics intra-communication (WAIC), used for radio-communication of safety and regularity of flight-related applications on board of a single aircraft, or the industrial, scientific, and medical (ISM) radio bands, whose use is generally limited to other applications. The Aerospace Vehicle Systems Institute (AVSI) project WAIC was formed in this frame to harmonise the regulations for on-board wireless communications. Based on an International Telecommunication Union (ITU) decision, a dedicated frequency band reserved for this purposed is in the 4.2–4.4 GHz range, to be operated in accordance with the recognized international aeronautical standards. It is based on short-range radio technology (<100 m) and maximum transmission power levels of 10 mW for low data rate, and 50 mW for high data rate applications [33,34]. Efforts are currently ongoing to make appropriate components commercially available for future applications. The ISM radio bands are, in turn, based on well-defined standards such as the IEEE 802.15.1 (Bluetooth), IEEE 802.15.4 (ZigBee) or IEEE802.11 (WiFi), where related COTS (Commercial of the Shelf) components are available. To demonstrate their feasibility, several SHM applications have been set up based on both these standards. As an example, autonomous SHM sensor nodes operating in the 2.4 GHz IEEE 802.15.4 ISM band have been developed for strain gauge applications, resulting in an average power consumption of 2.9 mW and a peak power of 49.3 mW in transmission mode [1]. Hence, considering the overall efficiency of the antenna with the transmitter, the peak power required for the low-power wireless connectivity can be estimated to be tens to hundreds of mW.

**Figure 1:**
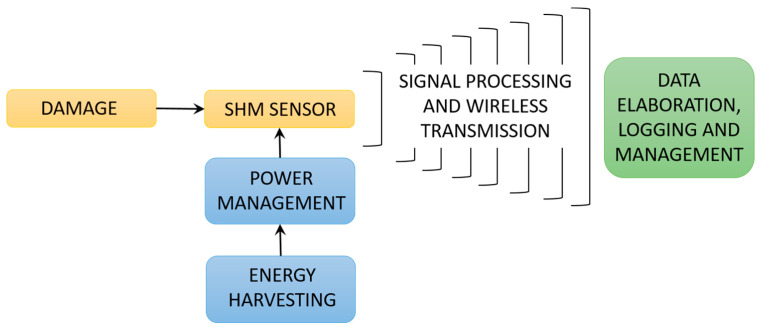
The ODIN SHM concept.

From the above treatise it is clear that low power consumption is one of the key enablers for introducing SHM sensors with interfaced data elaboration and wireless transmission modules into modern airplanes. In fact, the illustrated approach to SHM is progressively part of a trend towards autonomous sensor nodes for aerospace applications, and a growing research interest on their optimised deployment schemes [35]. An autonomous node is characterized in this frame by its ability not only to provide the initial signal processing of the raw measurement data to a format that can be compressed to a size suitable for fast and energy-efficient transmission to the data collecting unit, but also by the usage of the energy required for the operation of all the modules of the node in the form available in the environment surrounding it. The provision of this energy is therefore progressively being made by using energy harvesting (EH) technologies; the optimized use of this concept is therefore subject of thorough work in the framework of the mentioned EU COST Action ODIN, as represented in Figure 1. Although the sensing technologies themselves are not the focus of this work, the above analysis allows framing their importance for the aeronautical industry, as well as establishing the respective power requirements that could potentially be met by using EH principles.

**Figure 2:**
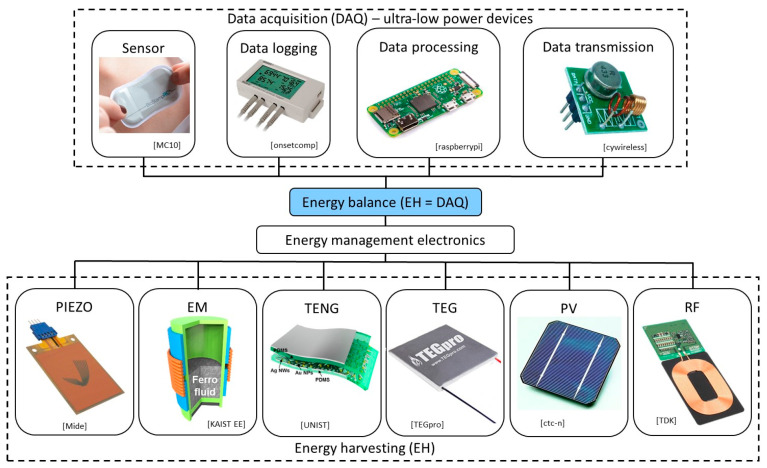
EH principle of powering autonomous sensor networks with the respective data elaboration and transmission modules.

In fact, EH-the process of collecting low-level ambient energy and converting it into electrical energy, is increasingly shown as viable means for obtaining autonomous sensor networks for SHM, communication, transportation, air and aerospace vehicles, robotics, MEMS devices, wearables, forest fire detection, and Internet of Things (IoT) components. Various sources of ambient energy and transducers are used in this frame (Figure 2): solar (and electromagnetic in general) radiation and photovoltaic (PV) generators, thermal gradients and thermoelectric generators (TEG), kinetic energy (induced by vibrations, by the deformation/strain of structures, by fluid flows and/or acoustic waves) and electromagnetic (EM), piezoelectric (PIEZO), or tribolectric (TENG) converters, and radio frequency with the respective RF transducers [6,36,37,38,39].

EH systems are increasingly shown to be also an effective power source for SHM systems and coupled peripherals used in aeronautic applications. In fact, according to the SAE international ARP6461 Guidelines for Implementation of Structural Health Monitoring on Fixed Wing Aircraft [40], EH systems, as well as electronic devices for data transmission, can be incorporated into SHM airborne equipment. The therein used EH systems (with their attachment/mounting interfaces) must, obviously, imperatively comply with safety standards in the given operating (i.e., mechanical, thermal, chemical, ice, dust, etc.) conditions, i.e., must not present hazards or have adverse effects on the aircraft structure or systems, must be reliable and long-lasting, cannot drain any energy, actively or passively, from the avionics systems or their power supplies, and should, ideally, require no maintenance for the useful life of the aircraft or the concerned parts. Thus, preference is given to harvesting elements which are applied externally to aircraft parts and may be replaced at will [40].

**Figure 3:**
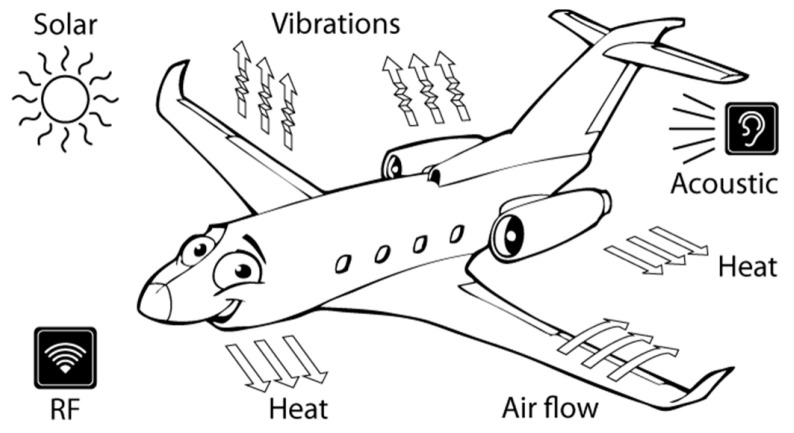
Scheme of an airplane with indicated energy harvesting sources discussed in this work.

In case of the herein considered fixed wing aircraft, due to their abundance on the airplane and some of its typical structural elements (Figure 3), mechanical vibrations (including that induced by the flow of air around the airplane) and thermal gradients, used separately or, to increase the possibility of providing the required energy constantly and reliably, simultaneously, are considered as the most promising sources for EH-based autonomous SHM sensor nodes [8,41,42,43]. Although some initial studies of integrated SHM solutions, combining advanced microelectronics with sensors, EH devices, power management, time synchronization, and web-based data distribution, have been proposed [44], due to the often varying nature of the power levels attainable from the physical energy sources in aircraft, as well as the above estimated needed power levels for an acoustic-based SHM sensor node with the coupled data elaboration and transmission modules, which could very well reach several hundred mW, a careful investigation of the available EH techniques and their applicability to the case considered herein has to be made.

The prior art aiming at providing a review of the usage of EH technologies for powering autonomous sensor networks used to monitor the structural health of mechanical components and machines is of limited suitableness in the herein considered case. In fact, existing reviews are either focused on application fields not related to aircraft structures [45], thus failing to take into account several important specific aspects, or, even when related to the aeronautical sector [42,46], they embrace a broad range of aircraft, without focusing on the fixed wing ones, they limit the given investigation to some of the available EH technologies, neglecting several of the rapidly developing ones, and they do not take into account the whole EH system with the related power management components. The aim of this work, carried out in the framework of the mentioned EU COST Action CA18203 ODIN [9], is hence to advance and broaden considerably the current state-of-the-art on the potential of all the diverse EH principles that could be used to power autonomous in-process SHM sensor nodes in the specific field of composite airplane components, updating it with the comprehensive review of the newest studies and principles suggested in literature. A careful analysis of the specific power outputs of the possible solutions, i.e., of the thus needed areas/volumes as well as of the excitation conditions (e.g., frequency or temperature ranges) is hence provided. What is more, a critical consideration of the needed power management and energy storage systems that would provide a stable power source for the considered SHM sensors and peripherals, in the presence of the dynamically changing excitation conditions, and/or while concurrently using several EH principles, is also provided. The other components of the ODIN COST Action will concurrently actively work on the optimization of the placement of the sensors, the minimization of the respective power consumption, and the optimization of the needed signal processing protocols as well as data transmission rates, which should all contribute towards decreasing the necessary power generation levels. The design guidelines derived in this work create thus an important element in determining the preconditions towards a strategic approach to integrate the outlined SHM principles already in the design inception phase of the airplane, thus considerably raising the technology readiness level (TRL) for a new class of autonomous sensor nodes for in-service SHM inspection of airplane components. These will then be materialized in the prosecution of the work on the ODIN EU COST Action CA18203, prospectively enabling the first generation of self-sensing aircraft capable of accurate structural prognosis [9].

In Section 2, Section 3, Section 4, Section 5 and Section 6 of this paper, a thorough review of the EH technologies to be potentially used to power autonomous SHM systems in airplanes will thus be given. The prospective usage of kinetic (vibration and strain—Section 2), TEG (Section 3), PV (Section 4), airflow and acoustic (Section 5), and RF EH systems (Section 6) will hence be comprehensively considered. The potential arrangements of state-of-the-art suitable power management electronics circuitry will be thoroughly reviewed in Section 7. Section 8 sums up the main findings of the investigation performed. The very broad list of the most recent references provided at the end of the paper gives to the prospective readers the possibility to deepen their understanding of all the aspects of the treated topics.

## 2. Kinetic Energy Harvesting Systems

Aircraft structures are subject to substantial dynamical effects during operation [38,47]. The thus exited vibrations are induced by both internal and external sources. The primary internal source of vibrations is the engine system, while air turbulence is the major external source of dynamical excitation of the airplane structure. The resulting kinetic energy is a viable source that can be transformed, by using energy harvesting devices, into useful electrical energy. This is generally achieved via the physical principles of electromechanical conversions, mainly electromagnetic induction, and the piezoelectric effect. Magnetostrictive devices, which use is associated with non-negligible cost and significant technological issues, have been subject to less attention, most often outside of aeronautic (i.e., higher frequencies) applications. Other EH principles proposed for kinetic environmental energy sources, such as electrostatic conversion [48], result in lower power outputs suitable more for MEMS and similar application fields, while their scaling up to the herein studied power magnitudes could be associated with considerable costs and technological challenges. The latter can be said also for the recently (last 10 years or so) proposed triboelectric nanogenerators [49], whose technological readiness is generally not yet on the levels required by the aircraft industry. These two classes of EH devices will thus be only shortly touched upon at the end of the treatise in Section 2.2 below.

The kinetic energy of dynamical excitation on airplanes could then be harvested by using either vibration EH devices, or by using the strain induced in the hosted structure [50]. In fact, the first study on the usage of vibration EH can be traced as back as to 1996 [51]. The main disadvantage of such an EH system, as will be further elaborated below, is that its optimal usage is generally limited to operating conditions in close proximity to one of its eigenfrequencies [32,52]. On the other hand, the potential for strain EH could be given by the large area of aircraft structures such as airplane wings [53]. The position and design of the EH devices are, in any case, crucial in obtaining efficient power generation. A series of vibration piezoelectric EH devices could, e.g., be optimally positioned on the airplane wing panel [47], whereas strain piezoelectric EH, referred to as multimodal smart skin, could be positioned in the area near the airplane window, providing a very interesting localized energy source for SHM applications [54].

### 2.1. Physical Principles of Electromechanical Conversion

As pointed out above, the most efficient and common kinetic EH devices are based on either the piezoelectric or the electromagnetic energy conversion principles.

In fact, in both vibration- and strain-based EH devices, the conversion of mechanical energy into electricity can be effectively achieved by making use of the piezoelectric effect, i.e.,

- In vibration energy harvesters the piezoelectric material is integrated onto an additional mechanical structure in the form of a mechanical resonator, thus inducing the strain of the piezoelectric material.
- In the case of strain energy harvesters, the piezoelectric material is, in turn, integrated onto the part of the airplane structure that during operation is subject to vibrations, varied loads, and similar dynamical excitation, inducing mechanical strain of the structure itself and of the piezoelectric material affixed onto it.

Electromagnetic energy transducers can, on the other hand, be used only as vibration EH resonators, as could maybe potentially also be the magnetostrictive EH devices.

#### 2.1.1. The Piezoelectric Effect and Operational Modes

**Figure 4:**
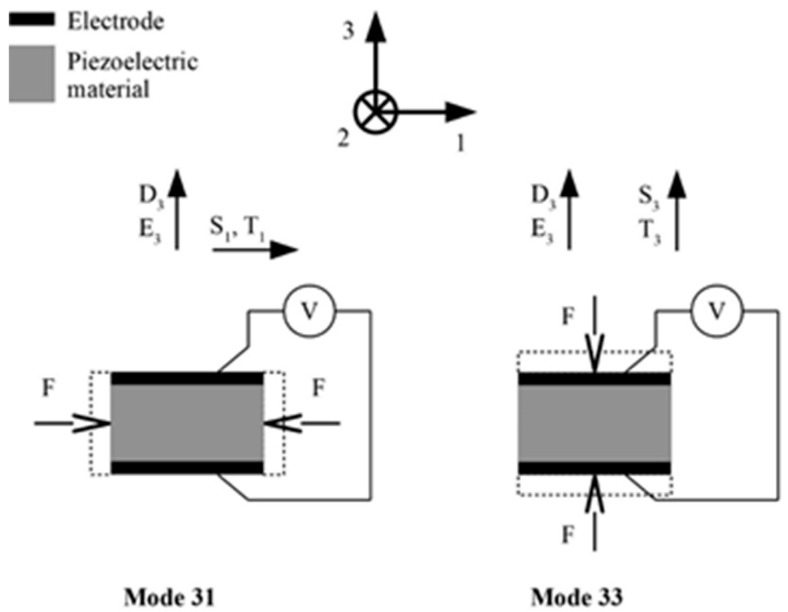
Operational modes of piezoelectric material for EH applications.

Piezoelectric materials have the property of converting, via the direct piezoelectric effect, i.e., via an electro-mechanical coupling effect, the energy of mechanical strains of the piezoelectric structure into electric fields (i.e., voltage), and, vice versa, via the reverse piezoelectric effect, convert electrical voltage into mechanical deformations [55]. The piezoelectric effect is expressed by constitutive equations allowing to determine it in six directions. Two modes, i.e., mode 33 and mode 31, are, however, the ones generally excited, and hence used in most of the developed EH applications (Figure 4). In both these modes the electric fields, and thus the generated voltage on the electrodes, are oriented along the polarization direction 3, while external forces induce strains along one direction only. In mode 33, this is the same direction as that of the voltage (direction 3), whereas in mode 31 the strains are along the perpendicular direction 1 [56].

#### 2.1.2. Electromagnetic Conversion

Electromagnetic induction, described by Faraday’s induction law, is the production of an electromotive force (EMF), i.e., of voltage across an electrical conductor in a changing magnetic field—the phenomenon that constitutes the foundation of electrical generators. When a permanent magnet is moved relative to a conductor, or vice versa, an EMF is created with the thus induced voltage being proportional to the strength of the magnetic field, the velocity of relative motion and the number of turns of the conductor coil. If the conductor is connected to an electrical load, current will flow, thus generating electrical energy, i.e., converting kinetic (motion) into electrical energy. This system is often proposed as effective means to attain kinetic EH systems, where the relative displacement of the permanent magnet with respect to the coil, caused by vibrations, is at the basis of the thus obtained kinetic EH transduction mechanism. A seminal work in developing autonomous monitoring and sensing in dynamically excited environments by using this principle is that of James and co-authors [57].

#### 2.1.3. The Magnetostrictive Effect

Physically somewhat similar to the piezoelectric effect, the characteristic property of magnetostrictive (often referred to also as ‘smart’) materials is that magneto-elastic coupling induces mechanical elongations if they are subjected to a magnetic field, while, inversely, their magnetization will change due to changes in applied mechanical stresses. The latter outcome, often indicated as the Villari effect, can be used in EH devices, where, however, an additional coil is necessarily used to obtain, via electromagnetic induction, electrical energy.

### 2.2. Vibration Energy Harvesting

**Figure 5:**
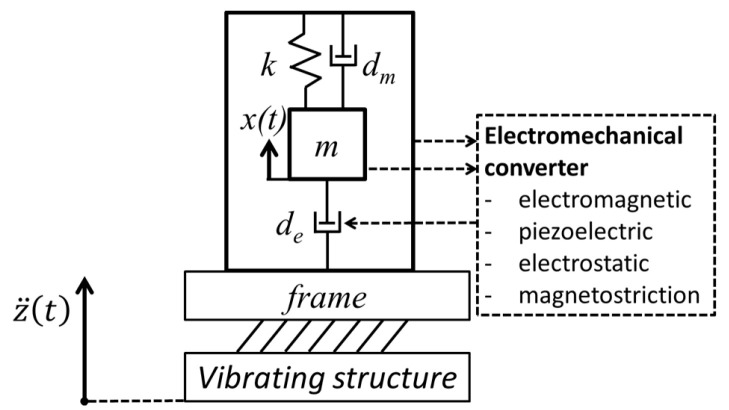
Scheme of vibration energy harvester [58].

The physical principle behind vibration EH systems is based on mechanical resonance and the electromechanical conversion of kinetic energy into electricity [52]. The thus used devices are based on either one end of the piezoelectric transducer, or else the permanent magnet (or the coil), being fixed to a frame, while the other element is attached to (or constitutes itself) the inertial mass of the mechanical resonator. Relative motion *x* of the movable seismic mass *m*, suspended on a spring (or a piezoelectric cantilever) with a tuned-up stiffness *k*, so that the resulting eigenfrequency matches that of the ambient mechanical vibrations, with respect to the frame, thus enables the kinetic EH transduction mechanism (Figure 5).

#### 2.2.1. Piezoelectric Vibration Energy Harvesters for Aeronautic Applications

**Figure 6:**
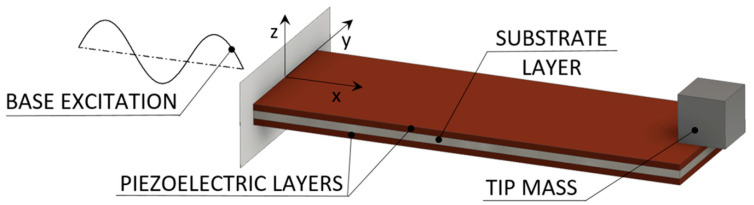
Piezoelectric bimorph vibration EH device [32].

Several designs of vibration EH devices for a broad application spectrum, depending, obviously, on the specific requirements and operative conditions, are proposed in literature [59]. Piezoelectric energy harvesting (PEH) vibratory devices, generally characterised by high output voltages, robustness, compactness, and light weight [42,60], are most common in the form of bimorph cantilevers with a tip mass placed on their free end, which amplifies the deflections and tunes the eigenfrequency of the device to the excitation frequency. By dynamically exciting the fixture of the cantilever, hence inducing the oscillation and the respective deformation of the piezoelectric layers, the electromechanical coupling effect results in an efficient conversion of kinetic into electrical energy. This results, via the above-mentioned operational mode 31, in the generation of electrical charge, i.e., induces a voltage difference between the electrodes deposited onto the surfaces of the piezoelectric layers (Figure 6) [61]. Given the electromechanical coupling effects (including the backward coupling phenomena), the behaviour of these devices is then assessed and optimised via complex numerical (i.e., finite element (FE)) models comprising modal, harmonic as well as linear and nonlinear transient analyses [32].

A major limitation, inherent in general to all resonant EH devices, is the narrow area of optimal operation around the eigenfrequency of the specific device. It manifests itself in a rapid decrease in output voltage and power values when the excitation frequency moves away from the eigenfrequency, limiting the possible application of such devices to specific cases with a constant excitation frequency [32]. This is particularly important when using PEH in environments with varying vibration frequencies, such as an airplane [38,62]. A suitable positioning of the EH device on the aircraft is therefore to be carefully chosen, taking into due consideration also the efficiency of the used power management electronics (see below). The placement on lightweight structures could, in general, be unsuitable, since the vibration EH device could affect the vibrations of the structure itself [63]. The possibility to mount the device in a spot with a stable and predictable dynamical excitation condition would, in turn, obviously, be preferable, so that the vicinity of the engine (e.g., its enclosure or its mounting fixture) would be a suitable and/or preferable environment—provided, obviously, that a suitable transmission of the thus generated energy to the considered integrated SHM systems, positioned along the airplanes’ wings and the fuselage, without introducing net weight due to wiring, is addressed.

In a study performed by Dunno and Batt on a twin-engine turbo propeller aircraft [64], it is concluded that the highest accelerations (with a magnitude of ~2.1 g) occur during ascent and descent, while a much smaller dynamic excitation level is detected at cruise altitude. Pearson et al. in [8] estimate the frequency range of the thus created vibrations to be from 0 up to 300 Hz, while in [65] Pearson et al. extended this range, for the rear edges of the aircraft wing panel, up to ca. 350 Hz. Dunno and Batt [64] also noticed that the excitation of the engines does not affect the rest of the structure, which is attributed to vibration absorbers built into the engine. If engine vibrations are to be considered as a source of kinetic energy, EH devices could, therefore, in addition to the mentioned engine enclosure or mounting fixture, be added to or integrated also into these vibration absorbers. On the other hand, due to the low Curie temperature, PEH devices cannot be used in regions of the airplane where temperatures can reach higher values, but also not at low temperatures where the piezoelectric effect decreases. The used piezo crystalline materials are also brittle and should thus be protected from impulsive loads.

In a detailed review of PEH technology, several types of PEHs, making use of operational modes 33 and 31, but also 15, in various design configurations, were analysed [66]. It is concluded that, in order to increase their efficiency, all PEH types would benefit from further optimization. According to the authors of this study, the operational mode 31 is the simplest in terms of fabrication and performance, while mode 15 is considered too complex, although exhibiting noteworthy potential for further research. Miniaturised PEH cantilever solutions, although potentially providing rather high maximal specific output powers (on the order of 10 mW/cm^3^), in absolute terms allow generally attaining only μW power levels, while their MEMS-related production can be technologically challenging [67,68].

In any case, several approaches to overcome the above evidenced limitation related to the bandwidth of operation, by broadening the optimal frequency spectrum, have been suggested in recent literature, the most promising being [32]:

- Changing the conditions around the cantilever free end (e.g., via damping control or active tuning), often inducing and/or combined with features inducing the nonlinear response of the PEH device.
- Changing the geometry of the PEH cantilever, herein including complex geometries with bistable configurations, or employing a large number of differently sized (i.e., tuned) cantilevers.
- Using a frequency up-conversion mechanisms, e.g., by plucking the free end of the piezoelectric cantilever and letting it oscillate at its eigenfrequency.

**Figure 7:**
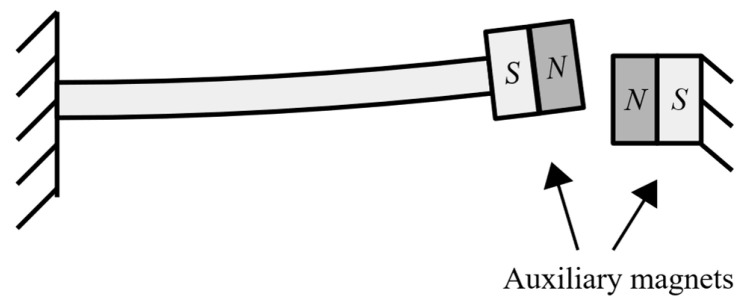
Topology of auxiliary magnets for nonlinear vibration PEH systems.

The process of changing the conditions around the cantilever free end usually comprises adding at the free end itself an additional system such as, e.g., a magnet (that could replace the therein foreseen tip mass), coupled to a coil mounted on the frame of the device, so as to enable, by influencing the axial stiffness of the cantilever, an active tuning of its dynamical response in a feedback loop [69,70]. In this regard these systems enter already in the realm of nonlinear stiffness devices, whose purpose, in general, is precisely that of extending the narrow frequency bandwidth. The addition of the extra active system at the free end increases, however, the complexity of the EH system, while, when a tuning coil is added, the system requires that a certain amount of the (generally quite limited) obtained electrical energy is used to power it. A variant of this system is the design proposed back in 2008, when a PEH cantilever with a magnet and coil system was additionally equipped with mechanical stoppers. The stoppers produced again a nonlinear response, i.e., a sharply hardening stiffness resulting in a wider dynamical response range [71]. Several other designs of cantilever PEH devices with auxiliary magnets (the above-mentioned coil is often substituted here by one or more permanent magnets opposing that on cantilever’s free end; Figure 7), inducing nonlinear stiffness, are proposed in available literature [72,73]. However, in addition to the mentioned technological complexity, such systems clearly demonstrate an additional potential drawback, i.e., the magnitude of the vibratory response amplitudes can vary considerably, which can be far from optimal for the considered EH applications in airplanes.

It is worth noting here also that, while the modelled response of the most common form of PEH cantilever resonators can be approximately linearized for specific operating conditions [74], this linear bimorph beam model can be extended to a nonlinear (coloured noise excitation) one by considering the influence of the forces of the permanent magnets used in the here described design configurations. The resulting load, causing a nonlinear stiffening effect influencing the obtained displacements, is also a significant source of additional potential energy for the EH system. In case of pronounced nonlinearities, the Duffing EH model could, in turn, be used [75].

**Figure 8:**
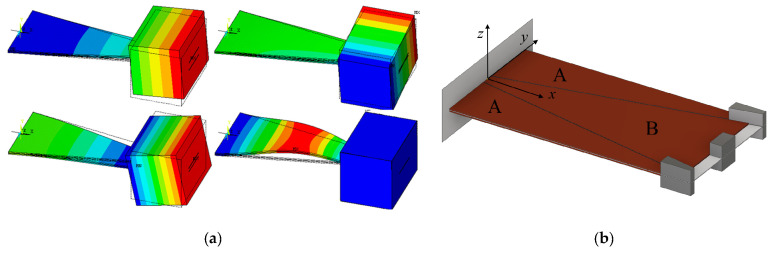
PEH geometry variations: optimized shape bimorphs proposed in [76]-reprinted by permission from Springer Nature (**a**) and segmented PEH geometry proposed in [32] (**b**).

Changing the geometry of the cantilever can constitute a viable and sometimes rather straightforward approach towards broadening the operational frequency spectrum or increasing the harvesting efficiency of PEH devices. Instead of a conventional rectangular shape, an optimized shape can hence be used, usually having the overall size that is the same, similar or at least comparable to that of the conventional shape. The shape can then be modified so that, while retaining the same dynamical response in the presence of a given excitation, much higher specific output powers are obtained by optimizing the strain (i.e., charge accumulation) in the piezoelectric layers (Figure 8a) [76]. The conventional shape can, alternatively, be segmented to comprise multiple PEH cantilevers optimized for specific electrical loads and possibly having a different eigenfrequency each, which can be tuned further by the proper choice of the respective tip masses (Figure 8b) [32,77]. The charge accumulation of these segmented PEH bimorphs can be enhanced further by ingeniously introducing stress concentrators or other ‘smart’ geometrical features [78]. These approaches do not require additional systems or excitation mechanisms, making them particularly suitable for the herein considered SHM applications, allowing to attain, in the optimal conditions, several mW (i.e., of the order of tens of mW/cm^3^) of output power.

Arc-shaped cantilever PEHs, having one or two semi-circular sections, have also been recently proposed [79]. For experimental purposes, these are compared to a standard flat harvester having either the same volume of the used piezoelectric material or the same length. It has been shown that, under an acceleration of 3g, the configuration with one semi-circular section provides more than 2.5 times higher output powers (i.e., >1 mW/cm^3^) than its flat counterpart, while the design with two arc-shaped sections provides even 3–4 timed higher output powers. It can thus be concluded that curved PEH cantilevers provide a significant increase in power output, at the expense, however, of the space (height) required for the installation of the elements. It is to be noted here that specific power equations, where generally the normalization is expressed in terms of the used harvesters’ material, do not consider the height required for the semi-circular elements.

Multiple stable states can, in turn, be achieved by using again complex PEH cantilever geometries or, as mentioned above, by employing magnets at the free end combined with stationary or additional oscillating magnets on the base of the device, resulting, once more, in a nonlinear PEH response. In the last 10 years or so, several studies on bistable and/or multistable piezoelectric cantilever beams with auxiliary magnets have thus been performed [80,81,82,83,84]. Although a wider frequency spectrum is indeed attained, potentially leading to higher power outputs, there is a conspicuous increase in the analytical complexity of the resulting strongly chaotic oscillations with large amplitudes, as well as a substantial increase of the volume of the resulting devices [85,86,87].

**Figure 9:**
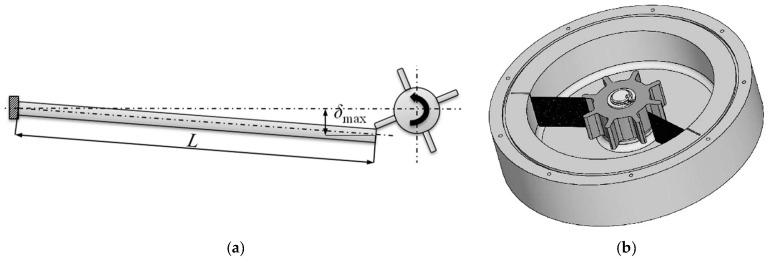
Scheme of the frequency up-conversion principle induced by plucking [92]-reprinted by permission from Springer Nature (**a**) and one of the thus proposed EH devices [32] (**b**).

Frequency up-conversion mechanisms (also referred to as impact driven mechanisms) allow, finally, the PEH cantilevers to always oscillate at their eigenfrequency, thus maximizing the output power regardless of the excitation frequency. The hence needed excitation can be provided by plucking the free end of the piezoelectric bimorph with a single plectrum or multiple plectra mounted on a flywheel or a moving mass (e.g., a moving ball or a mass on a spring), thus inducing the production of electrical energy during the ensuing mechanical vibrations (Figure 9). The principle was indeed proposed for an application in aircraft [88], although it is also applicable in other fields [32,89]. Such an approach requires, however, the addition of a separate mechanical excitation system, which can have a significant influence on the volume and complexity of the resulting EH devices, as well its price [90,91].

**Figure 10:**
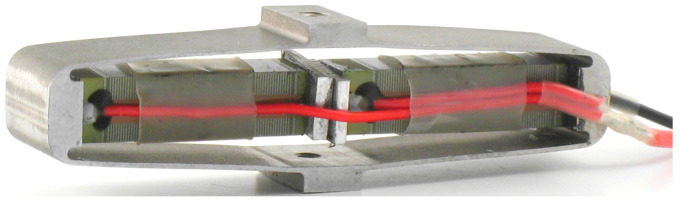
CEDRAT APA400M amplified piezoelectric stack (courtesy of Cedrat Technologies [93]).

Some of the proposed design configurations of vibration PEH devices are based, instead on using bimorph cantilevers, on piezostacks and on amplifying the stroke of excited oscillations of a seismic mass suspended via ingenious leverage mechanisms at the eigenfrequency of the resulting dynamical system, thus considerably enhancing the deformations of the stacks themselves. An example of such devices, available as off-the-shelf microgenerators, already certified for aeronautical applications, are those developed by CEDRAT Technologies [38,42], as shown in Figure 10. The generator, with a volume of 50 × 32 × 22 mm, exhibits a stroke of several centimetres, generating at 100 Hz, with a motion of the excitation mass of only 35 μm, 50 mW (i.e., 1.42 mW/cm^3^) of output power. Such modules can be combined to form larger stacks, preventing excessive voltage build-up, and installed, for example, inside the lag damper rods at the interconnection of the rotorcraft main rotor blades with the respective hubs (or the here considered aircraft’s engine vibration absorbers).

A much simpler PEH stack solution of this type, aimed originally at automotive suspensions but, with small adjustments, could also be applied to absorbers on aircraft engines, and comprising 3 piezoelectric stacks affixed to the base of a vibration damper (shock absorber), generated up to 85 mW of output power in laboratory settings [94].

Nonlinear PEH designs, also based on stroke amplification, but by employing piezoelectric plates or cantilevers between bow (cymbal)-shaped diaphragms with affixed seismic masses, were recently also proposed as a way to broaden the optimal frequency spectrum of operation [95,96]. Although generating rather large specific power outputs, these solutions are, however, quite complex and not yet considered as a viable solution for aircraft applications.

#### 2.2.2. Electromagnetic Resonators for Aeronautic Applications

**Figure 11:**
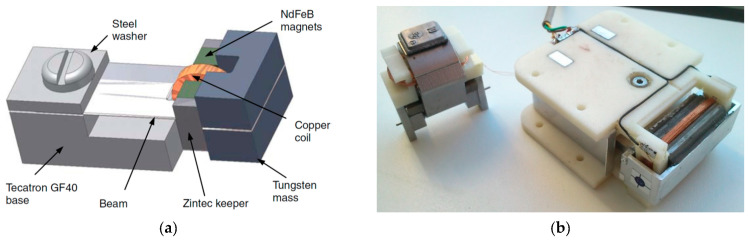
Miniaturized electromagnetic vibration EH devices proposed in [97] (**a**) and two versions of the ESPOSA EH devices [101] (**b**).

Pioneering work in the development of vibration EH with an effective electromagnetic circuit was performed by Beeby and co-authors in 2007 [97]. The thus proposed small (0.15 cm^3^) EH device, operating optimally at 52 Hz, allowed attaining a power output of 46 μW (a specific power output of 307 μW/cm^3^) (Figure 11a). A 50 x 32 x 28 mm electromagnetic power generator, that makes use of an aluminium alloy spring with NdFeB magnets, and produces 8 mW (0.18 mW/cm^3^) of power at 34.5 Hz and a 0.8 g acceleration is, in turn, proposed [98]. This device was also rearranged, based on the rapid prototyping design of the respective plastic frame, for a lower operational frequency of 17 Hz and, weighting only 120 g in a 120 cm^3^ envelope and generating a maximal power of 30 mW, has been successfully tested in rotorcraft assemblies [99]. A further solution applicable in helicopters, tuned up to a 28 Hz excitation frequency, and aimed exactly at powering health and usage monitoring systems, was developed more recently in the framework of the European FP7 project ESPOSA. A larger, 350 g variant, allowed attaining 85 mW of maximal power output [100], whereas 130 mW were obtained with the lighter 115 g version based on a moving magnetic circuit inside a printed aluminium frame [101] (Figure 11b). A test on a shaker showed that the latter EH device, when mounted in the tail area of a helicopter, could harvest continuously an average output power of around 16 mW. A helicopter health and usage monitoring system, powered by vibration EH, was proposed also by the AgustaWestland company [102].

The simplicity of all these electromagnetic EH devices, characterised generally by high output currents, robustness, and long lifetimes, at the expense, however, of low output voltages and potential problems due to electromagnetic compatibility [42], make them a likely candidate to be applicable, when scaled up to the needed few hundred mW power magnitudes, adapted to airplane vibrations as the excitation source, and placed appropriately in the identified zones near the aircraft engine with stable excitation frequencies, as the power supply element of the herein considered SHM systems with adjacent peripherals. This is further corroborated by the detailed review of more than 100 electromagnetic vibration EH devices for various application fields, some of which reached the commercial product stage, that is presented in [103].

#### 2.2.3. Piezoelectric vs. Electromagnetic Vibration EH Devices

**Figure 12:**
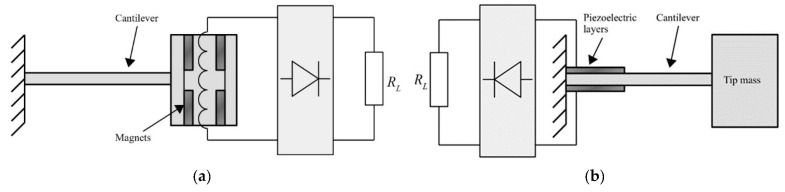
Model of electromagnetic (**a**) and piezoelectric (**b**) vibration EH systems for output power analysis [58].

The results of a thorough comparison of electromagnetic and piezoelectric vibration EH systems, with identical volumes, seismic masses, eigenfrequencies, quality factors, and excitation conditions, was recently presented [58]. Suitable mathematical models of both vibration EH device types were used for the calculation of the output voltages and powers (Figure 12), and, depending on the magnitude and frequency of the harmonic dynamical excitation, the recommended configurations of the most efficient vibration EH systems were determined.

**Figure 13:**
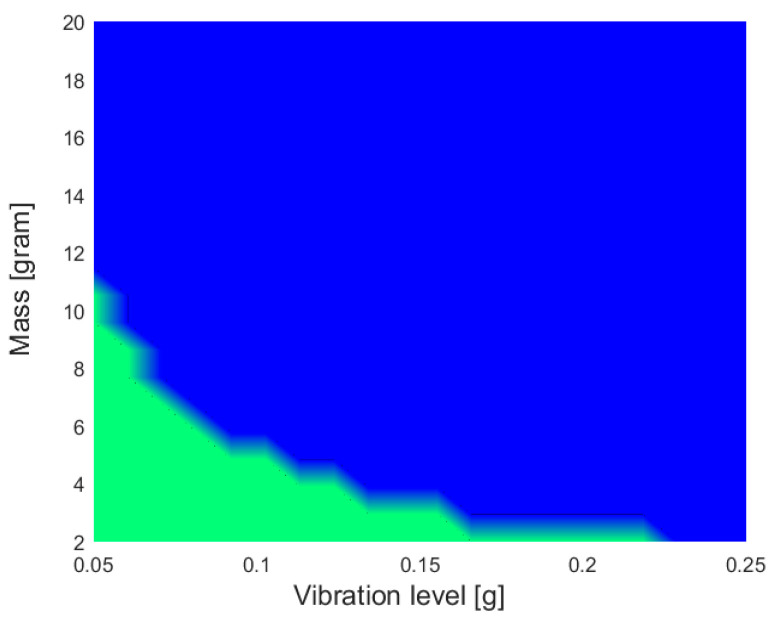
Simulation results: range of parameters where the piezoelectric harvester provides higher powers (green area) vs. that where the electromagnetic harvester performs better (blue area) [58].

The verified model of a commercial MIDE piezoelectric EH device was then compared with the electromagnetic system with the same characteristic parameters, operated at the maximal output power point as well. The model of the magnet and fixed coil, based on Faraday’s law, was integrated in the dynamical model of the resonator, allowing to determine the response of thus obtained electromagnetic system. It could therefore be shown (Figure 13) that the piezoelectric harvester is more efficient for applications with higher excitation frequencies (i.e., in the range from ca. 80 Hz upwards [42]) where, since the existing power management electronics have low efficiencies for voltages below 1 V, the electromagnetic systems provides low output voltages. On the other hand, in low excitation frequency conditions (in the range from ca. 25–80 Hz [42]), i.e., for systems with higher masses and higher motion amplitudes, electromagnetic systems can generate satisfactory output voltages.

#### 2.2.4. Potential to Use Magnetostrictive Vibration Harvesters in Aircraft

**Figure 14:**
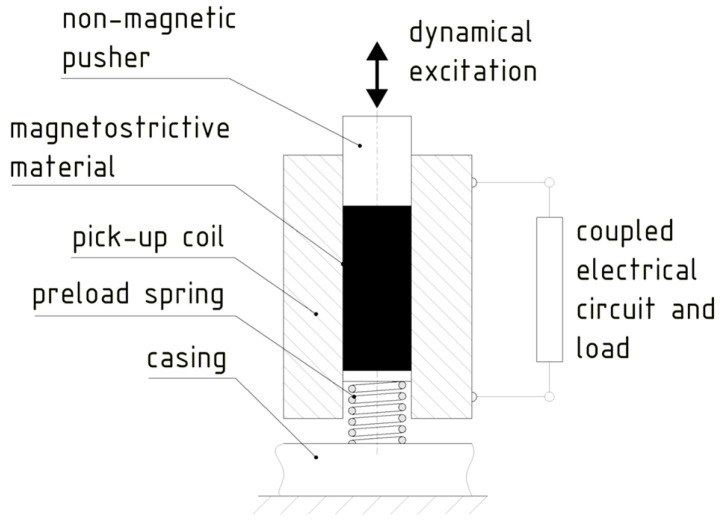
Typical magnetostrictive EH device.

As stated above, the potential usage of magnetostriction to attain, via the Villari effect, EH devices (Figure 14) to power SHM sensor nodes in aircraft, is coupled to significant technological challenges. The alloys displaying this effect, such as the most commonly used Terfenol-D (an alloy of terbium (Tb), dysprosium (Dy), and iron (Fe)), are, in fact, rather rare and thus related to higher costs. They are also characterised by a quite low magneto-elastic coupling (on the order of 10 μm/m for magnetic fields reaching the saturation level, as opposed to ca. 1 to 2 mm/m for the piezoelectric devices, or, vice versa, subject to large mechanical loads), allowing to obtain, in the optimized cases and often at higher excitation frequencies, roughly 1 mW/cm^3^ of maximal specific output power.

Given the above characteristics, magnetostrictive materials should be used where very high compressive loads are expected, so that the positioning of the device, such as on the engine mounts, is again a critical issue. What is more, it is shown that magnetostrictive devices are a poor choice for driving resistive loads, as the obtained power is inversely proportional to load resistance and the optimal load values are below 100 ohm [104,105,106]. Also, as already pointed out, the magnetostrictive rod has to be enveloped in a pick-up coil with, generally, a large number of turns, adding thus considerable weight to the already quite bulky system with magnetostrictive rods and permanent magnets (or another suitable pretensioning mechanism), while their output is nonlinear [107]. Last but not least, since magnetostrictive devices were originally developed and are still often used for military purposes, the data on some of their characteristics are often restricted [108].

#### 2.2.5. Potential to Harvest Dynamical Excitation Energy via Electrostatic and TENG Harvesters

Electrostatic EH devices transduce environmental energy via the capacitance change induced in an initially charged capacitor via vibrations. Although such systems generate high voltages, they perform well again only at their eigenfrequency, while, except if a series of differently tuned devices is not considered as a viable option, it is far from clear how to extend their operational bandwidth. Also, as already evidenced, their power outputs are in the tens of μW range, with the respective power densities at the level of tens of μW/cm^3^, while the MEMS-related production technologies used to manufacture them can be challenging with non-negligible cost, and the matching power management circuitry is complex [42].

In aircraft applications, an alternative EH concept, based on electrostatic charge built up in composite airplane structures during the flight due to the interaction between the airplane and the surrounding atmospheric environment, and its collection with capacitive collectors, a concept at the base of triboelectric (TENG) EH devices as well, was thus investigated in laboratory conditions showing that the electrostatic energy available on the aircraft can be regarded as a DC constant current source [109]. The subsequent study of the usage of this concept to power monitoring sensors in aircraft confirmed the possibility to collect and store a static charge in a capacitor, but also evidenced the very limited resulting energy outputs of 8 μJ [110]. The high amount of energy released via static discharging in-flight illustrates, however, the prospective potential of such a technology, also clearly indicating the need for its further investigation.

True triboelectric nanogenerators (TENGs), based on the relative motion (rubbing or impacting) of a dielectric on a metallic surface, result, in turn, in advantages such as high output voltages, high energy-conversion efficiency, abundant choices of materials, scalability and flexibility [111]. The to date reported power densities of harvesting vibration energy, in the frequency range from a few to a few hundred Hz, by employing TENG EH devices, are in the range of 0.04 up to, for 3D configurations, even 10.5 mW/cm^2^ [112,113,114,115]. TENGs technological readiness is, however, still limited by the used manufacturing processes and generally at the level validated in laboratory settings only, while no applications in aeronautic settings have been investigated. The power outputs of the 3D options could potentially be interesting for the herein considered applications though, so that the developments in this field should indeed be monitored and the proof of the prospective operation of such devices on airplanes should certainly be systematically studied.

### 2.3. Strain Energy Harvesting

As clearly evidenced above, the most widely used energy harvesters are based on piezoelectric materials. Due to their excellent sensing capability, these materials are often used also as sensors [24], although in this case several drawbacks can be evidenced, including the weight of some elements of the system as well as the wiring necessary for signal transmission. Piezoelectric patches and Macro Fibre Composites (MFC) are, in turn, piezoelectric structures that allow obtaining a flexible structure applicable to critical aeronautic components. A review of various flexible and stretchable piezoelectric devices for mechanical energy harvesting, sensing and actuation is given in [116]. The applicability of PEH devices, integrated onto the aircraft structure so as to transduce the therein available kinetic energy, inducing strain of the load carrying structure and, concurrently, of the piezoelectric element affixed onto it, into useful electrical energy, to be hence employed for powering the SHM sensor node with its peripherals, will be thoroughly elaborated in this section.

#### 2.3.1. Piezoelectric Patches and Macro Fibre Composites (MFC)

**Figure 15:**
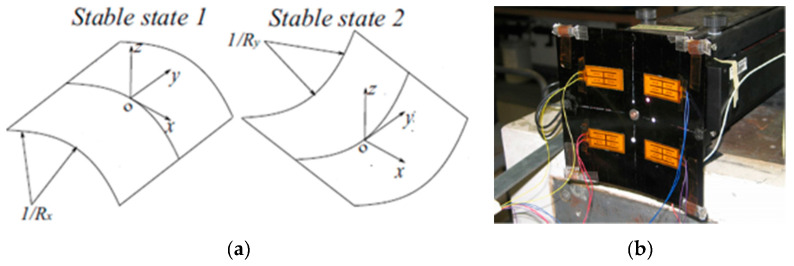
Scheme of a bistable carbon fibre plate (**a**) and PZT patches attached on the executed plate (**b**) [117]–reprinted by permission from AIP Publishing.

Four flexible piezoelectric strain patches, obtained by using the PZT-5A piezoelectric material, have been attached, in a suitably developed experimental set-up, to a bistable carbon fibre plate (Figure 15), observing hence the responses for both modes as well as for chaotic excitations [117]. The maximal measured power output at an acceleration of 2 g was 34 mW, while a clearly lower power output of 9 mW was measured in case of chaotic excitations. The simplicity of the proposed approach makes it quite interesting for possible aeronautic application, provided, obviously, that the evidenced wiring problem is properly addressed. The design, modelling, simulation, and test of a vibrating cantilever with bonded piezoelectric patches for EH and sensing purposes is, in turn, presented in [118]. A PZT-5A 30 × 30 × 0.2 mm^3^ piezoelectric patch, in a 2D strain loading condition, was considered in [119], allowing to attain at 25 Hz a maximal power output of 0.951 mW (~5.3 mW/cm^3^), with an optimal resistive load of 369.7 kΩ.

In the case of aeronautic structures themselves, in 2012 the performances of a strain PEH, made of a rectangular sheet of piezoelectric material housed in a flexible copper clad polyimide laminate, attached to a sheet of aerospace grade aluminium, excited with an EM shaker, intended precisely for aeronautic SHM, was investigated [8]. The resulting peak power output was 0.42 mW at 40 Hz and 1.1 mW at 300 Hz. The proposed approach could be applied on any part of the aircraft structure excited by vibrations from the engine, or from the turbulence or deflection of the structure itself during flight. The direct attachment of individual strain PEH patches on a model of airplane’s wing, making thus use of the inherent advantage of this EH methodology to be applicable on large aircraft structures, studied in a frequency range of 0–40 Hz, was, in turn, recently investigated, and a total energy output of hundreds of mW was obtained [120]. Importantly, it was also established that the proposed configuration allows substituting a 2 kg battery with the weight of the used piezoelectric material of 0.3 kg only. Finally, an optimization algorithm was recently used to find the optimal placement of commercial PEH devices affixed to a rectangular vibrating plate simulating an aircraft skin segment, i.e., the areas with the highest strain over the entire relevant frequency range [65]. The performed experimental validation has shown that, with respect to the originally positioned PEHs, the optimally positioned group of 4 PEHs allowed obtaining roughly a 50 % increase in power output, with a combined maximal output of 2.07 mW.

**Figure 16:**
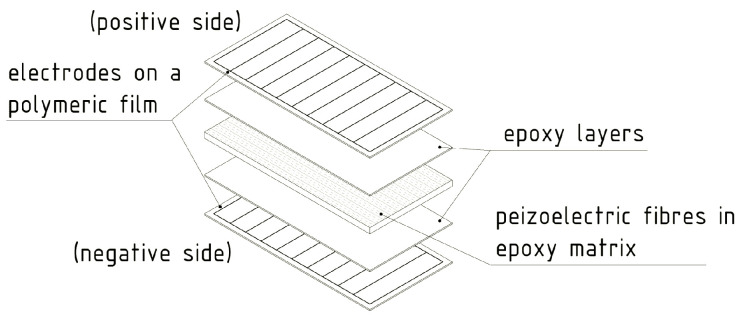
Generalised scheme of the structure of an MFC transducer.

MFCs are, on the other hand, transducers developed by NASA, particularly for SHM applications. Their structure is made from thin unidirectionally aligned piezoelectric active fibres, sandwiched in a polymer matrix between two sets of electrode patterns (Figure 16). This composite structure was tested in loading modality 31 on an active area of 85 × 28 mm^2^, having a 0.3 mm thickness, and with a 5.44 g/cm^3^ material density, with cyclic peak-to-peak strain loading of 600 με at excitation frequencies of 2, 4, 6, 8, and 10 Hz, and then at 10 Hz with strain loadings of 300 με, 400 με, and 500 με. In the best case (strain loading of 600 με at 10 Hz), up to 3.34 mW (i.e., 0.14 mW/cm^2^, 4.7 mW/cm^3^, and 0.86 W/kg) were produced, while the energy delivered over 60 s was 200 mJ [121].

An optimal modelling of MFC structures in the frequency domain, and the prediction of the hence harvested power, is very important in adapting this EH technique to airplane structures. A vibration EH prototype, with MFC patches bonded to a cantilever beam, was hence fabricated, the respective output voltage was calculated using an FE model, and the performances have been verified experimentally [122,123]. The modelling of MFC structures is presented also in [124]. A recent theoretical and experimental study of the response of piezoelectric patches with interdigitated electrodes, connected to double curved flexible shells, is, in turn, presented in [125]. The dynamical performances of the therein considered MFC patch is investigated using frequency response functions and compared to FE modelling results of the respective electromechanical response, resulting in a good agreement.

**Figure 17:**
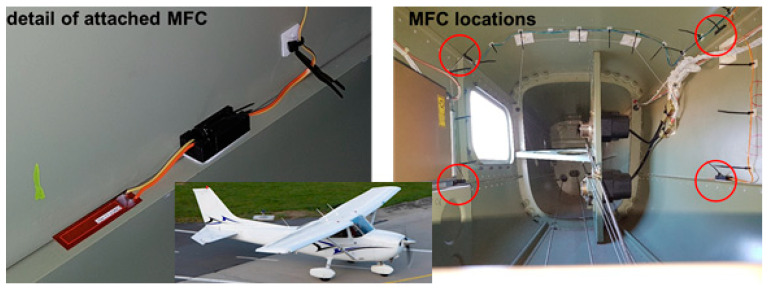
MFC patches embedded into the structure of a small civil aircraft [29]–reprinted by permission from Elsevier.

The power output of four MFC PEH devices on a vibrating aluminium plate, aimed exactly at the herein considered case of powering SHM systems on aircraft, was, in turn, tested in [126]. The EH device was configured in this case as a skin mounted harvester that, due to its simplicity, could be incorporated in an existing aeronautic SHM system. Since the maximal measured power output was 36.3 mW (i.e., 0.7 mW/cm^2^ and 3 W/kg), it was concluded that, depending on specific requirements, a PEH area in the range from 71 cm^2^ up to 5.1 m^2^ would be necessary to power a typical SHM system. In the case considered in this work and in the ODIN Cost Action, an area of roughly 0.07 m^2^ would, therefore, suffice to meet the needs of an AE or wave propagation SHM sensor node with the respective data elaboration and wireless transmission modules. The test of MFC EH patches, embedded into the structure of a small civil airplane to directly measure the response related to structural vibrations (Figure 17) was presented in [29]. The functionality of the entire measurement chain was tested during an experimental flight, and the acquired data are used for further research of data-processing, impact detection, and structural health predictions.

#### 2.3.2. Polymer Piezoelectric Skin and Composite Structures

The design methodology of strain piezoelectric EH devices that are tightly encapsulated into the structural (load carrying) components and exploit the higher order eigenmodes, which could be excited on the surfaces of airplane elements (i.e., wings, horizontal and vertical stabilizers, fins, flaps, and similar components), often referred to as piezoelectric or smart skin systems, and mentioned already in the Introduction of this work as means of achieving SHM [30,31], is described in [127]. These systems could, hence, act synergistically and concurrently as both SHM sensors and EH sources used to power them [53,54].

In order to further reduce the weight and size of PEH skin systems in airplanes, thus minimizing their influence on the aerodynamics [30], piezoelectric polymers and porous polymer systems [128] are increasingly considered as a very promising approach to the herein foreseen purposes. From the EH perspective, among these materials, those showing the most promising electromechanical coupling performances when subjected to dynamical excitations, are thin polymer sheets based on poly(vinylidene fluoride) (PVDF) [129] and polypropylene (PP) [130]. These sheets can be obtained by large-scale fabrication techniques, such as extrusion or blown sheet extrusion, allowing rather thick (ca. 100 mm) structures, advantageous from the practical point of view, to be obtained. Despite having more favourable characteristics than other piezoelectric polymers and allowing to attain high output voltages [92], pure PVDF is also characterized by low electromechanical coupling [131]. To improve the overall efficiency of the PVDF PEH systems in direct applications as part of airplane wings, composite structures comprising an active filler, with an overall density below 2.5 g/cm^3^, could, thus, be a promising approach. AlO-rGO systems could be used in this framework since, due to their strong dielectric characteristics, they provide additional dipoles, potentially, hence, doubling piezoelectric coupling [132]. There are indications that the modification of graphene oxide in PVDF formation, by using poly(methyl methacrylate) (PMMA) atom transfer radical polymerization, could also significantly improve the energy harvesting capabilities, although generating a relatively high-weight structure [133]. In other recent studies, alternative possibilities to enhance the harvesting efficiency of PVDF-based PEHs, based on advances in the nanotechnologies, are thus investigated. Using electrospinning to obtain PVDF nanofibers, modified with PMMA and single-walled carbon nanotubes (SW CNT), permit thus attaining a high porosity (lightweight) structure allowing, at an excitation frequency of 50 Hz, an electrical output of 3.11 V (four times larger than that of an equivalent structure in PVDF alone) [134]. On the other hand, a promising concept could also be the employment of a PVDF composite foam obtained via a physical foaming agent and using a multiwalled (MW) CNT piezo-active crystalline phase, resulting, for a 1.5 wt. % of MW CNTs, in output voltages of 6.5 V (ca. 6 times higher than that of an equivalent structure in PVDF alone) [135]. The possibility to use polyethylene (PE) PEH films to convert strain energy, induced by gusts (air turbulence) vibrations of aircraft wings, into electricity was, in turn, investigated in [88], showing that specific power outputs of the order of 1 mW/m^2^ can be obtained.

A strain wireless sensor node, powered by using MFC EH patches attached onto a carbon fibre composite material that emulates a section of the aircraft skin, and aimed at SHM, was alternatively proposed in [136]. An EH application for aircraft composite structures, using MFC principles, was studied also in [137], where the developed co-curing, used to integrate the EH elements into the carbon-fibre composite airframe structures, offered, if compared to those obtained by direct bonding methods, harvested powers higher by up to 23%, with maximal attained power values, for low frequency excitations (1-100 Hz), between 0.16 and 42.1 mW. An overview of the key elements of various manufacturing techniques, of the respective work-flow steps, as well as of the factors impacting the performances of the final products, all used to obtain smart composite structures with embedded piezoelectric transducers was recently provided in [138].

### 2.4. Summary of Findings on Kinetic Energy Harvesting

Based on the above lengthy analysis of kinetic EH devices, it is clear that vibration energy harvesters could very well be used as part of the airplane engines’ enclosures and respective mounting fixtures, be tuned to the respective operational frequencies for maximum efficiency, and result in rather high specific output powers, thus efficiently powering the herein considered SHM systems with the adjacent peripherals. The performed analysis, as well as the already mentioned SAE international ARP6461 Guidelines [40], allow also evidencing that, due to their power density, simplicity and versatility, piezoelectric or MFC devices, and strain energy harvesting devices in general, mounted on the aircraft skin or structural members, could be a very suitable solution for harvesting energy in aircraft applications. This class of strain devices also allows easy access for maintenance or parts’ replacement and, provided that suitable integration technologies are used and all the required safety aspects are met, could, also considering their low thickness, provide solutions that would not significantly affect the aerodynamic properties of the aircraft.

Teams developing the hence proposed kinetic EH systems refer to the resulting output parameters with a variable degree of detail, so that generally the comparison of the different proposed design configurations is not straightforward. For the purposes of benchmarking and comparing the overall performances of energy harvesters, different metrics are hence proposed in literature. The power density, i.e., a specific power value in terms of the maximal output power normalized with respect to the volume (or, for planar structures, the area) of the EH device, such as mostly reported above, can be a proper parameter for strain-based devices but cannot, in general, be considered as a suitable indicator for vibration EH systems, since it does not take into proper consideration the value of the excitation acceleration. Effectiveness, perhaps the first of the thus proposed comparison parameters for vibration EH devices [139], did not address satisfactorily this shortcoming. Normalized power density (NPD), where power density is divided with the square of the acceleration magnitude, was hence proposed [97] (Table 1). A further improved metric, which takes into account also bandwidth aspects of vibration EH, is designated as the Figure of Merit (FoM) [140]. An alternative form of the FoM, based on the quality factor, was also proposed [141], but this metric does not correctly embrace the outputs of non-linear systems for wider bandwidths. A quite complex metric for vibration EH devices is, finally, the normalized power integral density (NPID) [142]. NPID includes outputs for different excitation frequencies, and the power integral is used for its calculation. The resulting intricate calculation reflects also input acceleration, the volume of the device, and the total energy in the considered frequency bandwidth. Obviously, in the case of aeronautic applications, power densities normalized to the mass of the devices would also be an extremely useful metric, but the masses of the devices proposed in literature are reported very seldom. No definite indications can thus be deduced in this regard.

The kinetic EH devices proposed in literature and reviewed above allow then establishing that PEH vibration devices, generally in the form bimorph cantilevers, allow attaining peak output power densities *PD*, when the devices are matched to the respective electrical loads, in the range of several mW/cm^3^. Although data necessary to calculate the respective normalized power densities *NPD* values, as the most immediate comparison metrics taking into account the excitations as well, is seldom available in reported literature, from the available data it is possible to deduce that maximal *NPD* of roughly 0.07 mW/(cm^3^·m·s^−2^) can be obtained with this class of devices. The respective maximal specific power levels normalised to the mass of the device could, very roughly, be estimated to be in the 0.3 mW/g range. The advantage of PEHs is the already broad spectrum of proposed solutions allowing to extend the frequency bandwidth of operation, although some of them via quite complex configurations. Configurations with larger bandwidths would, in turn, allow to use the PEHs not only in the vicinity of the airplane engine, but also in other dynamically excited components such as the wings, the horizontal and vertical stabilizers, the fins, the flaps, and similar components. PEHs are also characterized by high output voltages, robustness, compactness, light weight, and suitability for higher excitation frequencies.

In the case of electromagnetic resonators, peak *PD* values of several hundred μW/cm^3^ can be achieved, implying that rather big respective device volumes (and resulting masses) are needed for the SHM airplane applications considered in this work. This class of EH devices allows, in turn, to attain maximal *NPD* values by an order of magnitude higher than that of PEHs, making them the preferred choice for applications where low frequency and high amplitude vibrations at higher excitation levels are expected, while the space constrains are not so stringent. What is more, they are characterised by simplicity, high output currents, robustness, and long lifetimes, at the expense, however, of low output voltages and potential problems due to electromagnetic compatibility.

Magnetostrictive devices, although also allowing peak *PD* values in the mW/cm^3^ range, are characterised by maximal *NPD*s by an order of magnitude lower than that of the PEH devices, as well as by the evidenced significant technological challenges. The technological issues are limiting the applicability of electrostatic and TENG EH devices as well, while they allow attaining *PD*s of, respectively, tens of μW/cm^3^ (with the corresponding *NPD* of roughly a μW/(cm^3^·m·s^−2^)) and from 0.04 up to, for 3D TENG configurations, 10 mW/cm^2^. What is more, the internal impedance of TENGs is very high, which is generally hard to match with most of the commercially available electronics characterised by low impedance.

In the case of all the considered kinetic EH principles based on dynamic excitations, fatigue lifetime considerations have to be also carefully considered so as to achieve a reasonable mean time between failures (MTBF).

The strain EH devices, piezoelectric patches and MFC solutions are shown to be potentially attractive since they could be placed on large areas of composite aircraft structures themselves, such as the airplane wings or the mentioned other dynamically excited structural airplane components, but also because they could be concurrently used both as EH solutions and as SHM sensing elements. These devices allow obtaining again maximal *PD* values of several mW/cm^3^, whereas polymer piezoelectric skin solutions enable attaining maximal *PD*s of the order of only 0.1 μW/cm^2^.

## 3. Thermoelectric Energy Harvesting Systems

As already evidenced in the Introduction of this work, next to mechanical (kinetic) energy, reviewed at length in chapter 2, thermal gradients are also a very abundant energy source and thus often considered as a viable and valued possibility to develop EH systems in airplanes. Although thermoelectric energy generation based on radioisotope thermoelectric generators (RTGs) is well known in aerospace applications on spacecraft for decades, thermoelectric energy harvesting on aircraft relies generally on exploiting precisely the spatial and/or temporal thermal gradients. In order to convert thermal into electrical energy, thermoelectric generators (TEGs), that exploit the available temperature differences between two surfaces, are hence frequently considered in the herein studied framework. Test flights have proven the potential of TEGs in providing viable power outputs coupled to long-term stability, but further research work is needed to achieve higher TRLs for their factual integration in aircraft. In the following the respective state-of-the-art is thus thoroughly reviewed.

### 3.1. Working Principle of the TEG Module

**Figure 18:**
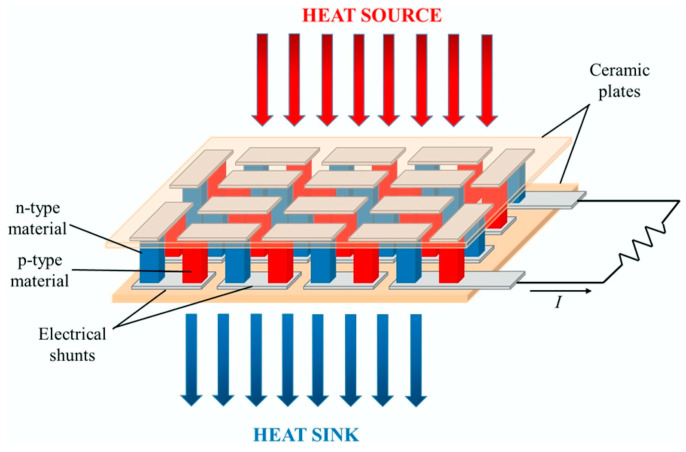
Scheme of thermoelectric power generation [146]-reprinted by permission from Elsevier.

A TEG module is placed between a hot (heat source, at a temperature $T_{hot}$) and a cold (heat sink, at a temperature $T_{cold}$) side, converting the temperature difference ΔT and heat flow present in a suitable region of the aircraft (see below), via the Seebeck effect, into a DC electrical power supply. TEG modules consist of p- and n-type semiconducting materials (legs) connected electrically in series and thermally in parallel (Figure 18). The p-type elements have holes as the charge carriers and, when there is a heat flow, a current flow is generated in the same direction. On the other hand, the n-type elements have electrons as charge carriers so current and heat are counter flowing. The thermocouples are electrically connected in series, so that the voltage generated by each leg pair (thermocouple) is added to get the total voltage produced by the TEG, while the current through each pair is the same [143,144,145].

The main criterion for the selection of thermoelectric (TE) materials/legs is their Figure of Merit (FoM) *ZT* defined as:

(1) $$ZT=\frac{\alpha^{2}\cdot \sigma \cdot T}{\kappa }$$

where *α* is the Seebeck coefficient, while *σ* and *κ* are, respectively, the electrical and thermal conductivities at the absolute temperature *T*.

The temperature dependence of *ZT* makes each TE material category suitable for different applications, based on the respectively considered temperature ranges. In the herein considered case of EH in airplanes, materials such as those based on bismuth telluride Bi_2_Te_3_ may commonly be used, having a *ZT* value of about 1 in a broad range between room temperature and ~200 °C. Other materials (i.e., silicides) are very promising for applications at higher temperatures, being at the same time lighter, which, as already pointed out, is a very important parameter in aircraft.

**Figure 19:**
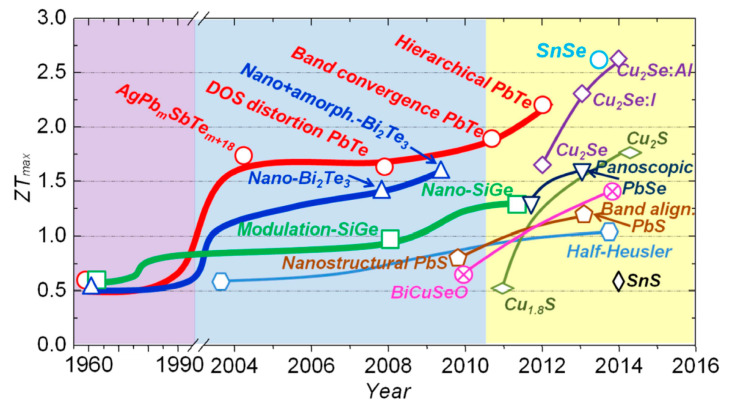
Chronological progress of the FoM *ZT* for different TE materials [147].

**Figure 20:**
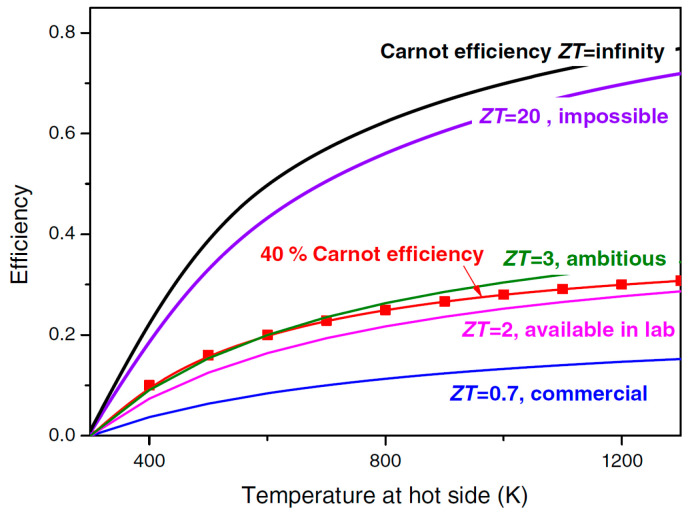
Conversion efficiency of TE materials vs. ΔT (for $T_{cold}=300K$) and *ZT* [148]-reprinted by permission from Springer Nature.

Although commercially available TE materials have the FoM value of about 1, research on the development of new materials brought the *ZT* values to magnitudes higher than 2.5 (Figure 19) [143,147]. The influence of *ZT* on the conversion efficiency shows, however, that, to ensure that the TEG efficiencies become competitive to that of traditional power generators, which can reach 40 % of the theoretical Carnot efficiency, the FoM value of the TE materials should reach at least 3 (Figure 20) [148]. The currently available *ZT* values are still lower than that, so that the reachable power densities of TEG devices remain limited, and, although flexible TEG modules are being developed, the available devices are still rather bulky, rigid and brittle, as well as not always easy to manage in environments with high temporal fluctuations of temperature. The interest in thermoelectric EH devices is, however, justly very strong. In fact, they are a result of a quite mature technology, and characterized by autonomy, robustness (no moving parts involved), no emissions, long operational lifetimes proven for decades in spacecraft missions, no maintenance requirements, as well as commercial availability with dedicated EH electric circuitry already often included [42,60].

**Figure 21:**
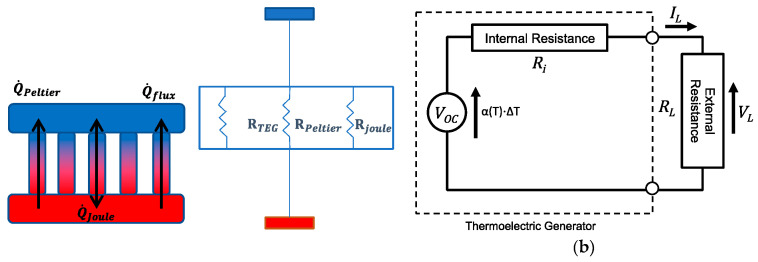
Model of TEG heat fluxes with equivalent thermal (**a**) and electrical circuits (**b**) [149].

In order to determine the maximum power output of a TEG module, all the involved crucial TE effects are then to be considered. In Figure 21a is hence depicted a 1D thermal model of a TEG along with its thermal circuit equivalent. The total TEG thermal resistance can thus be calculated from this model and used in the thermal analysis, allowing to determine the temperatures on the two sides of the EH device. The equivalent electrical model (Figure 21b), including the internal resistance and voltage source of the TEG module, with the attached load resistance affecting the optimal operation of the device, is then used to study the resulting electrical behaviour of the TEG module [149].

The electrical power output is calculated as the product of the current $I_{L}$ through the load resistance $R_{L}$ and the respective voltage $V_{L}$:

(2) $$P_{el}=V_{L}\cdot I_{L}=I_{L}^{2}\cdot R_{L}$$

The voltage source and the total resistance determine the current flowing through the circuit:

(3) $$I_{L}=\frac{V_{OC}}{R}=\frac{\alpha \left(T\right)\cdot \Delta T}{R_{i}+R_{L}}$$

where $V_{OC}=\alpha \left(T\right)\cdot \Delta T$ is the open circuit (Seebeck) voltage, *α*(*T*) is the Seebeck coefficient, while $R_{i}$ is the internal resistance. Then, substituting Equation (3) into (2), the resulting expression for the electrical power output $P_{el}$ is thus obtained:

(4) $$P_{el}=\frac{{\left(\alpha \left(T\right)\cdot \Delta T\right)}^{2}}{{\left(R_{L}+R_{i}\right)}^{2}}\cdot R_{L}$$

Maximizing the temperature difference ΔT across the TEG is, therefore, the key element for maximizing the respective electrical power output of a TEG. The maximal TEG efficiency is then defined as [143,150]:

(5) η(ΔT)=ΔTThot·1+ZT−11+ZT+TcoldThot

Due to the limits of the currently attainable *ZT* values evidenced above, a single TEG module cannot produce sufficient electric power for the herein considered SHM sensor node with the adjacent data elaboration and wireless transmission peripherals. Several modules are, therefore, used in order to achieve the necessary output power levels. TEGs are hence connected electrically in series/parallel in order to reach the required voltages and currents. This setup, along with suitable heat sinks and thermal connections, thus creates a thermoelectric generator system that, given a certain temperature difference ΔT between its two sides, can deliver the required electrical power to the load.

### 3.2. Thermoelectric Energy Harvesting in Airplanes

EH systems based on the usage of TEG systems in airplanes offer significant cost benefits for both manufactures and airline companies. In fact, as already evidenced above, the reduction in manufacturing cost is the result of reduced cabling of autonomous remote wireless sensors or passenger accessories, which, in turn, reduces airplane weight and thus lowers fuel consumption, which is a benefit for the airlines [151,152].

In an airplane during flight, different ranges of temperature differences can be found at different locations. Depending on the thus available ΔT, the potential of energy harvesting, as well as the power output ranges, vary considerably. The magnitude of ΔT can, therefore, be used to group TEG EH modalities in three main categories, which are systematically described in the following subsections.

#### 3.2.1. High Temperature Difference Applications of TEGs in Airplanes

Modern airplane jet engines are becoming more energy efficient, although there is certainly room for further improvement. The generation of electric power by using TEG modules, that can be installed on the hot surfaces of a jet engine nozzle, is certainly part of this efforts. In fact, the gases in the nozzle reach temperatures exceeding 620 K, while the temperature of the bypass flow around the nozzle can be as low as 240 K. A recent study demonstrated through modelling and simulation that the implementation of a larger number TEGs in these conditions can produce an electric power as high as ca. 1.65 kW per engine. With higher nozzle surface coverage and improved heat sink design, the obtained power output could be increased to even 3 kW [153]. An approach where exhaust gases were used to increase the temperature of the hot side of the TEG module was recently experimentally investigated on a jet engine as well [154].

**Figure 22:**
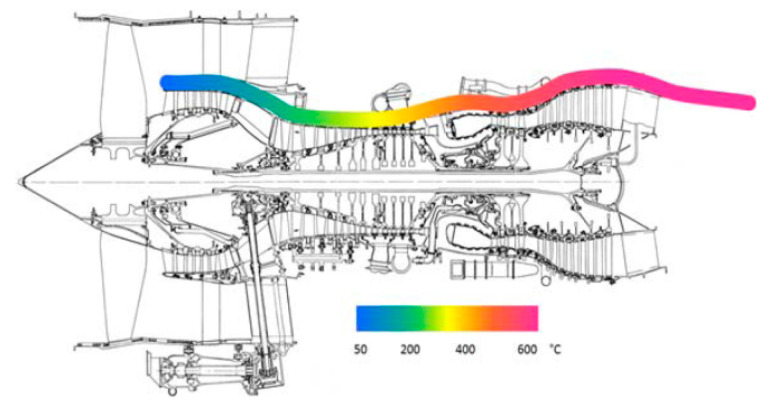
Typical casing temperature profile along an aeroengine with high bypass ratio [155].

The potential of using TEGs in aircraft jet engines in positions close to components such as the high-pressure turbine, the low-pressure turbine, the nozzle or one of the exhaust ducts, where temperature gradients are high enough for the most efficient TEG functioning, was studied also in [155]. A reference aircraft model with an advanced technology level for an entry-into-service in 2030 was defined, with a geared turbo fan with an unmixed nozzle designed for it and set as a reference engine. Starting from a conservative and standardly achievable TEG efficiency (*η* = 8 %), first calculations, based on the temperature profiles obtained from the simulations of the turbine (Figure 22), have been performed in combination with analytical and FE-based performance analyses for the TEGs. Given that the available temperature gradients and power densities vary significantly along the engine, there are multiple locations that could then be used for positioning the TEGs. During airplane’s cruising, TEG operation allows hence attaining promising results so that, using heat pipes for thermal transport, specific powers from about 1 kW/m^2^ at the nozzle to ca. 9 kW/m^2^ at the high-pressure turbine, could be obtained. Due to the limited available space and other installation constrains, the deployment of the TEG at the nozzle seems, however, to be a technically more viable solution. The performed analyses confirm, in any case, that, under the given geometrical conditions, the specific power requirement could be fulfilled on the module-level, and TEGs might therefore be used to at least support the conventional generator.

**Figure 23:**
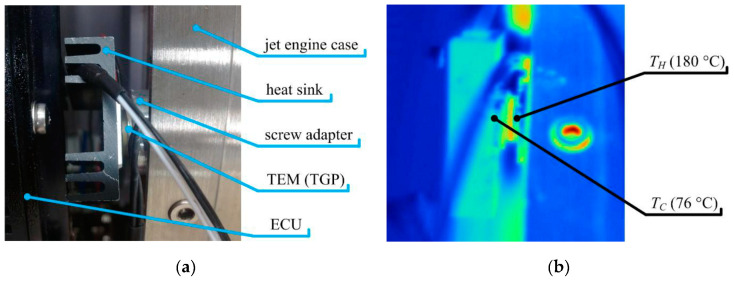
TEG demonstrator (**a**) and thermographic image of its location (**b**) [157]–reprinted by permission from Elsevier.

An autonomous SHM system based on crack sensor is, in turn, described in [156]. In this case, the power is supplied by using a dynamic TEG harvester at temperatures of up to 200 °C with Eythritol as the phase change material (PCM). Energy outputs of about 40 J were thus attained, which was proven sufficient for assuring the monitoring of cracks that could be induced in the aft pylon fairing area by hot gases exiting the jet engine. A concept of a backup TEG for aeronautic applications was then tested on a proof-of-concept technology demonstrator on a small airplane turboshaft engine TS100 (Figure 23). It was thus shown that the backup electrical power source, based on a MEMS TEG module with suitable power management, power conditioning and energy storage elements, might be used only in the failure state of onboard power lines of the aircraft, providing the needed electrical power for various low-power systems [157]. High temperature differences can also be achieved at the nose tips of supersonic aircraft, where the airflow around the tip can significantly increase the temperature. Using computational fluid dynamics and the thermal conduction theory, it was hence shown that ΔT values of up to 757 K are developed for supersonic flights at Mach 4.5 [158]. Small Unmanned Aerial Vehicles (UAVs) are, in turn, typically powered by two-stroke internal combustion engines characterized by a high power-to-weight ratio. These engines have, however, a low chemical-to-mechanical energy conversion efficiency of about 12%, so that a significant amount of energy is generally wasted via the exhaust gases. Part of this energy can, thus, be harvested by using TEG modules installed on a canister-like modified engine muffler mounted outside the UAV, while the hot side of the TEGs is suitably interfaced with the high temperature exhaust gases. After a design optimization phase, more than 40 W of net electric power was hence obtained from the 455 K exhaust gases of a Bat4 UAV [159].

#### 3.2.2. Medium Temperature Difference Applications of TEGs in Airplanes

**Figure 24:**
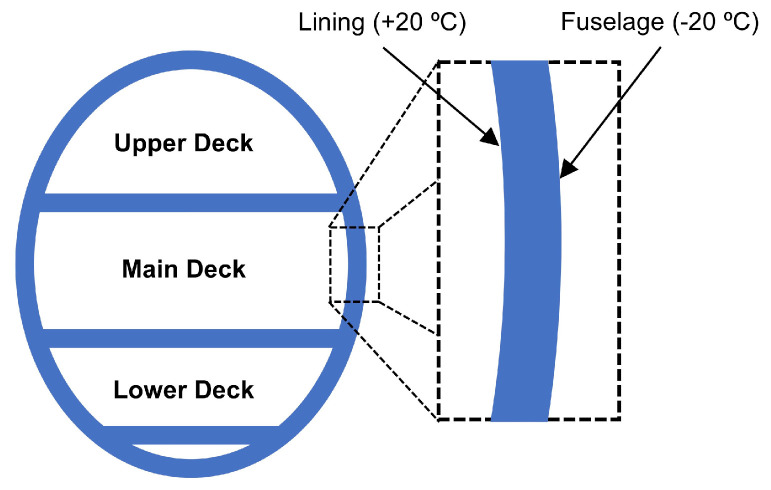
Possible installation of TEGs in airplanes’ fuselage lining [162].

During operation, the fuselage of airplanes is subjected to significant thermal gradients, with outside temperatures in the range of about −50 °C (with, obviously, large outside temperature variation during take-off and landing) and cabin temperatures of about +20 °C. In practice, due to the aerodynamic friction of air with the surface of the aircraft, the temperature on the outer surface of the fuselage is higher than that of the external environment, reaching thus ca. −20 °C to −35 °C [149]. In any case, the resulting medium ΔT values of ~50 °C can be achieved in the space between the airplanes’ fuselage outside surface, serving as an “infinite” thermal energy well, and the internal wall of passengers’ cabin (Figure 24). This provides an opportunity for the application of TEGs as EH devices, already considered in this context as a viable means for powering SHM systems [1,8,150,160,161].

**Figure 25:**
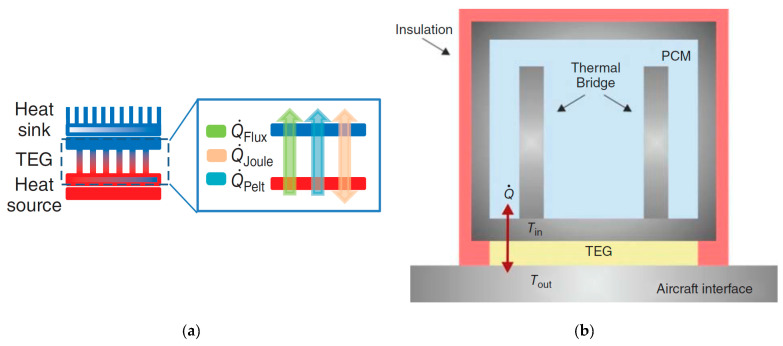
Static (**a**) and dynamic (**b**) TEG configurations used in airplanes’ fuselages [150].

A “quasi-static” and a “dynamic” state approach to harnessing the therein available energy can then be defined. In addition to TEGs, in the “quasi-static” state approach only passive components (i.e., heat pipes and/or sinks) are included in the generator, while in the “dynamic” state approach thermal masses, increasing the thermal inertia, are included to maintain the required ΔT between the two sides of the generator during takeoff and landing (Figure 25). In the latter case, the thermal capacity is often increased by using the latent heat of phase-change materials (PCM) [150].

**Figure 26:**
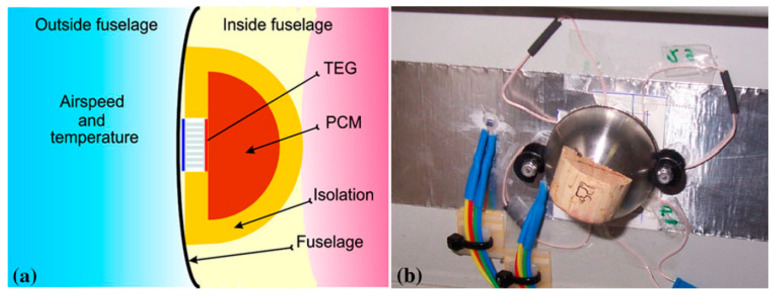
Scheme of the TEG mounting (**a**) and its factual installation close to the front left door on a DL-R A320 D-ATRA aircraft (**b**) [170].

A TEG making use of a PCM heat storage was hence designed, simulated, and tested suggesting that an autonomous aircraft power source, feeding the sensor nodes of SHM systems, is possible [163]. An improved TEG with adapted capacitors in the power management system allowed then attaining an improvement of the performances by ca. 50 % [164]. The effect of using various PCMs on the performance of TEGs was, in turn, investigated, by using water as reference, via tests on groups of organic and inorganic materials [165,166,167,168]. The first flight results with such thermoelectric harvester, used to realize aircraft-specific wireless sensor nodes, indicated reliable operation, while experimental results, compared to predictions from theoretical models, demonstrated that the developed simulation models can be used to consistently predict the power output of this class of EH devices [169]. What is more, the results of 28 flight tests during a 6-month flight campaign, with flights categorized based on flight duration at the maximum altitude and on ground temperature, with two identical TEG EH devices mounted on the aircraft (Figure 26), were analysed in terms of significant operational parameters such as energy output, maximum altitude, and fuselage temperature, showing a strong correlation between flight altitude and minimum fuselage temperature [170].

**Figure 27:**
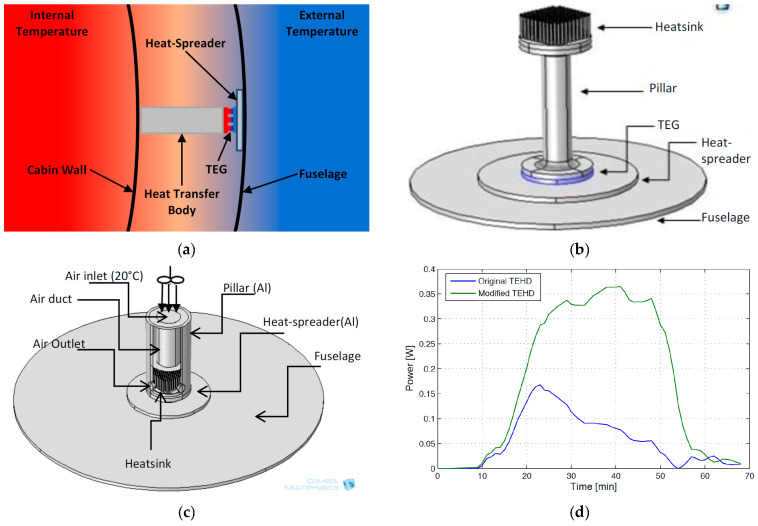
Scheme of the TEG mounting on the fuselage (**a**), initial harvester design (**b**), modified harvester design (**c**) and output power obtained for both design configurations (**d**) [162].

Using, in turn, the “static” state approach, a TEG harvester mounted on the fuselage was also studied (Figure 27a). In the initial EH device design (Figure 27b), the heat sink was positioned at the top of a high conductivity aluminium pillar aimed at maximizing ΔT across the TEG, i.e., bringing the fins of the heat sink closer to the cabin environment; this pillar configuration can also support various ancillary electronics and energy storage components. The cold side of the TEG was in contact with an aluminium plate, known as the heat spreader. Since a sufficient power output could not be attained with this design configuration, a modified design, where the bulky pillar is eliminated and the heat sink is in direct contact with the TEG so as to reduce the thermal inertia, was introduced (Figure 27c). In this configuration the system resulted in a maximum output during take-off and landing, i.e., when the fuselage temperature was changing faster relative to the time constant of the TEG itself. The obtained maximal power output was, therefore, more than doubled (Figure 27d) [162].

The enhancement of TEG performances by developing an air-cooled heatsink for low-power wireless SHM applications was, then, investigated in [171]. To allow for the TEG reaching the wireless SHM operational power requirements of, in that case, tens of mW and, at the same time, adapting the assembly to the complexity of aeronautic SHM arrangements, a hybrid heat diffusion system, composed of copper heatsinks and highly oriented pyrolytic graphite layers, was, hence, proposed. The thus obtained experimental results allowed establishing that the developed arrangement generates a power output on the level of 25 mW. Different options for autonomous SHM systems in airplanes powered by using TEG EH devices were reviewed in [172].

#### 3.2.3. Low Temperature Difference TEGs on Airplanes

Energy harvesting from passengers’ bodies is one of the main ideas behind the employment of low temperature difference TEGs in airplanes. Although the power output is low, the potential for self-sufficient operation of low power wireless devices, as an alternative to onboard power sources, is an attractive perspective. The integration of TE devices in airplane seats was, hence, suggested in this framework as means to supply the power required by wireless sensors embedded in the seats themselves. In fact, infrared images of airplanes seats allowed clearly revealing hot spots corresponding to the thigh and kidney/back area. The resulting heat was then used via TEGs to achieve the target power value of 1 mW necessary for operating a wireless sensor system in a demonstrator, thus enabling an automatized reporting of information on chair occupancy, armrest or tray table position etc. [173].

Very recently, further experimental studies of similar arrangements showed that the average maximal temperature produced from passenger’s body can reach 35.5 °C. A resulting temperature difference ΔT of 15.5 °C is therefore obtained, so that, by using a standard TEG, maximal power outputs at the level of 10 mW are generated [174]. A factorial study aimed at determining the influence of characteristics such as passengers’ age, gender, weight, and height was also performed using statistical models, showing that these factors do not have a significant effect on the obtained ΔT values, whereas the ambient temperature is, obviously, found to be influential [175]. Similar ΔT values as in the latter case can be consistently expected also on the hydraulic pipelines along the fuselage of the airplane or on the wastewater system, allowing to attain, by using Micropelt high-tech thin film TEGs, peak output powers of up to 8 mW. A single TEG of the same type, positioned on the cargo skin (where ΔT≈40 K), generated, in turn, up to 34 mW of maximal power output and average power outputs of ca. 22.6 mW [8]. It is important to note in this regard, especially in the case of the herein considered low output harvesting applications, that the obtained maximal powers correspond to load resistance values $R_{L}$ that match the internal resistance of the TEG itself, i.e., that a careful matching of the TEGs and the foreseen electrical loads is imperative. What is more, the output power will be relatively high in a narrow area around this peak, decreasing rapidly for both higher and lower $R_{L}$ values, although the decrease is more rapid for $R_{L}$ values lower than TEG’s internal resistance [176].

Last but not least, novel integrated concepts of using concurrently several TEG EH systems in various applications and temperature difference ranges in aircraft are being considered by BAE Systems Military Aircraft and Information (MAI) at Warton. These applications could include aircrew clothing with integral novel TE materials/devices within the fabric, supplementary electrical power generation from propulsion jet pipes using TEGs, and incorporation of TEGs within heat exchangers to enhance system performances [143].

A short note should finally be devoted here also to the pyroelectric effect, i.e., the property of certain dielectric materials that show a spontaneous electrical polarization as a function of temperature, generating high voltages with good dynamical bandwidths. Some pyroelectric-based EH solutions have been proposed in literature as an alternative to TEGs in environments where temperature changes more in time than spatially, but rather little has been established so far on such materials, their conversion efficiency is low, while the respective manufacturing costs are high. No applications in aerial vehicles (or even in commercially available devices) have thus been shown so far, pending further progress in the MEMS technologies that constitute the basis for the advancement of such devices [42].

### 3.3. Summary of Findings on Thermoelectric Energy Harvesting

The above review on the usage of TEG EH devices, shows clearly the increasing interest of using such rather mature and commercially available technology in airplane applications. In fact, high ΔT values, found along the aeroengines, could enable attaining realistic *PD*s as high as 100 mW/cm^2^, allowing potentially the usage of TEG-based EH technology in powering the herein considered integrated autonomous SHM wave propagation sensors with adjacent data elaboration (signal processing and data logging) and wireless transmission modules. In this regard, a suitable transmission of the energy from the areas around the aircraft’s engine to the SHM systems positioned along the wings and the fuselage, without introducing net weight due to wiring, should again be properly addressed. The usage of TEG at medium ΔT values, generally foreseen in the airplanes’ fuselage between the outside skin and the lining, could constitute a very interesting alternative, whereas lower level temperature differentials can be considered as an EH source at the interfaces to the hydraulic and waste-water or ventilation systems or even, due to the heat released by human occupants, in the passenger cabin itself. In these latter cases maximal absolute output power levels of the order of tens of mW can be obtained from a single TEG, while the reachable energy conversion efficiencies are still generally limited. The development of flexible TEGs could, in turn, allow alleviating the potential issues related to their rigid and rigid and brittle structure. A possible interesting development of TEG EH devices for on-the-spot integration using 3D printing was then recently also presented [177]. The application of pyroelectric EH devices, due to their low conversion efficiencies and high costs, is currently not considered a viable option for applications in aircraft.

## 4. Photovoltaic Energy Harvesting Systems

Having addressed at length in Chapters 2 and 3 the two most abundant energy sources, and, thus, the most studied EH principles to be applied for powering SHM systems in aircraft, namely the kinetic and the thermoelectric EH modalities, in the following sections the possible employment of other prospective EH technologies for the foreseen application, that generally attract far less attention in literature, starting with photovoltaics, will be reviewed as well.

In fact, during their operation airplanes are exposed to solar radiation and provide, especially in the case of the herein considered fixed wing airplanes, and similar in this regard to the above-described strain EH devices, large surfaces that can support photovoltaic (PV) conversion technologies. Although solar energy harvesting has not been investigated as a means for powering SHM equipment in aircraft yet, research on solar-powered airplanes and supplemental solar power supplies is actively being carried on as means of using renewable energy and decreasing the environmental impact of aircraft.

The first solar-powered airplane performed its maiden flight already in 1974 [178]. A considerable number of large-scale projects have then led to significant developments in unmanned and manned solar-powered airplanes [179,180]. Despite the fact that solar-powered airplane designs are generally striving at a different goal, typically requiring novel designs from the ground up, the findings made in this domain over the years are of considerable interest to evaluate the potential for solar-powered SHM systems. Some of the most relevant studies in this domain include those of Ramirez-Diaz et al. [181] and Liscouet-Hanke et al. [182], who investigated the potential for PV systems to be used as a power source to, at least partly, meet the electrical energy needs of commercial airplanes during operation. These studies show that the properties of PV devices, the external influences, and the potential integration of the technology into an airplane all have to be properly considered.

### 4.1. Photovoltaic Cells and Systems

**Figure 28:**
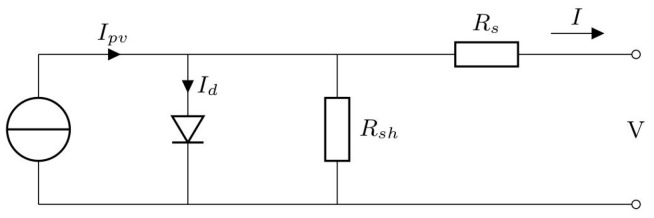
Diode model used to describe the behaviour of the PV cell.

PV cells convert solar irradiance into electrical energy. On illumination, the incident radiation creates in the semiconductor device electron-hole pairs, thus inducing output voltage and current. The behaviour of PV cells can be described quite accurately based on a simplified diode model (Figure 28) that results in the following current-to-voltage relationship:

(6) $$I=I_{pv}-I_{0}\left[exp\left(\frac{V+R_{s}I}{nV_{t}}\right)-1\right]-\frac{V+R_{s}I}{R_{sh}}$$

where *I*_pv_ is the photocurrent of the PV cell, which has an approximately linear relationship with solar irradiance, while *I*_o_ is the diode reverse saturation current that has a strong temperature dependence. *I* is the output current of the PV cell, *R*_s_ is its series and *R*_sh_ the respective shunt resistance, *V* is the terminal voltage of the cell, and *n* is the diode ideality factor. Finally, *V_t_* is the thermal voltage, defined as

(7) $$V_{t}=\frac{kT}{q}$$

where *k* is the Boltzmann constant, *T* the absolute temperature of the p-n junction, and *q* is the electron charge.

**Figure 29:**
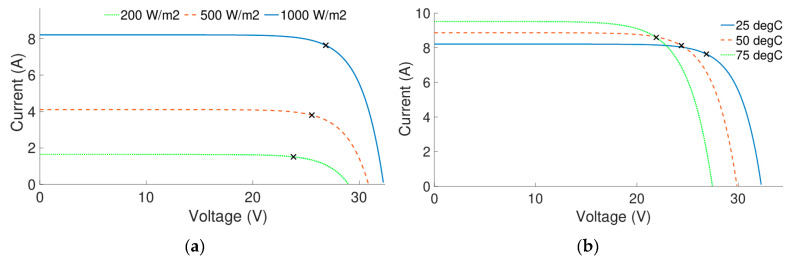
*I*-*V* characteristic of the PV cell vs. variation in irradiation (**a**) and temperature (**b**).

The PV cell’s *I*-*V* characteristics on Figure 29, qualitatively illustrating the effects of changes in irradiation and temperature, highlight three distinct points: *I*_sc_–the short-circuit current observed at a terminal voltage of 0 V, *V*_oc_–the open-circuit voltage, i.e., the maximal achievable terminal voltage (that also implies a null output current), and the point *V*_mp_, *I*_mp_–where the highest levels of power extracted from the cell are achieved (indicated in the Figure with a cross mark on each of the shown curves). The characteristics depicted in Figure 29 allow also evidencing that a change in the irradiation has large effects on the output current (with larger irradiation leading to larger *I_sc,_ I_mp_* and *V_oc_*), whereas an increase in temperature leads to an increase of *I_sc_* and *I_mp_*, but also to a reduction of *V_oc_*.

**Figure 30:**
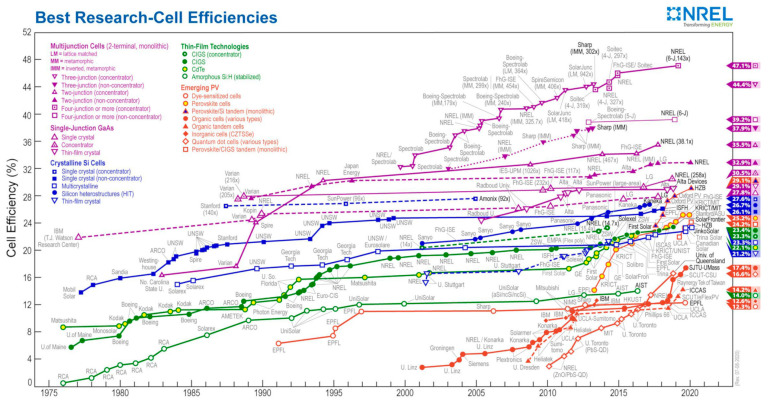
NREL chart of highest confirmed conversion efficiencies for research PV cells (courtesy of the National Renewable Energy Laboratory, Golden, Co. [183]).

Based on the used PV technology, different PV cells demonstrate varying conversion efficiencies. The conversion efficiency may thus vary from a few percent for thin-film PV cells, up to even 45 % for recent multi-junction technologies (Figure 30). Besides conversion efficiency, in the selection of the technology appropriate for a determined application, cost issues and the weight of the substrate need however, to be considered as well. The PV technology mostly utilized up to date in commercial systems is crystalline silicon, demonstrating conversion efficiencies of approximately 20–25 %. In order for PV devices to result in feasible output voltages and currents, for the majority of applications, multiple PV cells need to be combined in a panel. Series connection of cells results here in an increase in voltage, whereas parallel connection induces an increase of the output current. Besides the PV panel, a PV system contains also an output regulator, that provides a stable power output to the load, and, to enable the operation of the PV panel as close as possible to the point of maximum power output, it often integrates a maximum power point tracker. Next to energy management electronics, and due to the intermittent availability of the energy source, in most application scenarios, an energy storage device is essential to provide a buffer for shorter or longer periods of low/absent solar radiation (see also Section 7 below).

### 4.2. External Influences

In most applications, including aircraft, a challenge concerning the usage of PV systems is the dependency of their performance on many external parameters [184,185]. In fact, the expected output power is dependent on environmental factors, such as temperature, humidity, wind, and cloud coverage, as well as operational parameters such as geographical location, time of the day, day of the year, direction, and tilt. Although a number of these parameters can be considered, with more or less effort, in mission planning [181,182], an uncertainty and mission dependency remains. A potential PV system to be used for powering SHM sensor nodes in airplanes will, consequently, likely need to be over-dimensioned, particularly for most application scenarios in commercial flights.

One fundamental uncertainty is the estimation of available solar radiation, which is the basis for the assessment of the available energy during the flight. Aglietti et al. in [186] present a model for the estimation of solar radiation at different altitudes, based on the fact that at higher altitudes the sunbeam crosses a thinner atmospheric layer, while pressure levels are lower and cloud coverage can be expected to be significantly smaller. It is demonstrated that, at altitudes between 6 and 12 km above sea level, 5–10 times more annual irradiation can be expected with respect to that at ground level. The study is, however, limited to one geographic location and does not consider the motion of the airplane.

A number of studies [181,182,187,188] make, in turn, use of models that take into account the movement of the airplane, and estimate the solar radiation on a surface of the airplane during a given mission. To evaluate the amount of energy that can be harvested under realistic conditions, a number of scenarios can then be evaluated, including flight and parking at different geographical locations and on different days. The obtained results demonstrate that energy levels of the same order of magnitude as that required by onboard electronics can be harvested by using a PV system [181,182]. These studies are, however, limited to rather long flights, and specific routes or locations. What is more, for the energy balance to be successful, large areas of the airplanes need to be covered by PV cells, while economical or engineering aspects of their integration are generally not investigated.

Although it is clear that temperature has a significant effect on the performances of PV devices (see also Figure 29b above), and that the efficiency of a PV cell drops with increasing temperature, due to the involved complexity [184], the effects of temperature on the performance of PV devices in aircraft are neglected in many of the available studies. In fact, the estimation of the PV cell temperature is quite a challenge since it will raise due to the incident solar irradiance, but at the same time the surrounding air temperature can be assumed to be cold and additional convective cooling is induced by the air flow. In many cases the effect of temperature is, thus, considered negligible with respect to other uncertainties, and the efficiency is supposed to be similar to that at room temperature.

### 4.3. Summary of Findings on Photovoltaic Energy Harvesting

The above treatise allows evidencing that, when compared to many other energy harvesting sources (see above and below), harvesting of solar energy is a well-developed technology that provides a high specific power density on the level of up to 140 mW/cm^2^ (i.e., a patch of ca. 2 × 2 cm^2^ could meet even the most demanding requirements of the herein considered SHM sensor with the adjacent data elaboration and wireless transmission peripherals) in a lightweight structure [60,189]. Due to the high altitude at which many airplanes operate, and many relatively flat areas they provide, PV technologies are thus considered as an interesting option for power generation. Studies of solar-powered airplanes have demonstrated that sufficient energy can be generated to operate on solar energy alone, and over extended periods of time, small unmanned and manned aircraft [178,187,190,191,192]. Other studies have demonstrated that solar power has the potential to supply the needed electricity on commercial planes during the entire flight, which is of the order of hundreds of watts [181,182].

Based on these results, it is not a question whether PV technologies on airplanes can supply sufficient amounts of energy for the herein considered SHM systems with connected data elaboration and wireless transmission modules or not, but rather how such a solution could look like and whether it is economically feasible and reliable. In fact, as pointed out above, commercial flights may occur in different weather situations, at different geographical locations, with different flight lengths, and during different times of the day. A solar-powered SHM system on a commercial airplane would thus need to be able to operate in any of these conditions. In contrast to solar-powered airplanes. Moreover, it is not feasible to change flight paths, times, or logistics in order to optimize the conditions for solar-powered SHM implementations. Consequently, these systems would have to be designed for worst-case scenarios and, to support potentially long periods of low solar radiation, could imply the need to install also relatively large energy storage reservoirs (with their respective masses). A viable alternative is to combine PV with some of the other illustrated EH technologies, optimizing with suitable electrical circuitry their parallelized operation in changing environmental conditions so as to maximize the resulting power outputs.

The integration of a solar-powered SHM system on a commercial airplane is another challenge to be addressed. While most solar-powered airplanes are designed from scratch, a solar-powered SHM application, if not foreseen already in the design inception phase, could require retrofitting the system on an existing aircraft design, which implies determining a favourable placement of the PV cells while avoiding negative effects on aerodynamics and weight [181,182]. On the other hand, while solar-powered airplanes require high conversion efficiency in order to generate enough energy from limited aircraft surfaces, in the case of solar-powered SHM applications, requiring relatively small powers, lower efficiencies may be acceptable. This opens up possibilities for new PV materials that may be integrated more easily, or even combined solutions with the used aerofoils [193]. In any case, specific research aimed at gaining a better insight into the opportunities and challenges of solar-power integrated SHM systems in airplanes is yet to be conducted.

## 5. Airflow and Acoustic Energy Harvesting Systems

The energy of the air flowing around the airplane is also a practically unlimited well of environmental energy to be transduced, via EH technologies, in useful electrical energy. Its effects were in principle already considered in the above Section 2 dealing with kinetic environmental energy, especially in the case of strain EH system whose excitations are mostly induced by deformations of airplane structures induced by the dynamical effects caused by airflow. Two other EH principles based on using airflow energy, increasingly investigated in literature very recently (i.e., in the last ca. five years or so), will, in turn, be considered in this section as potential power supply sources for the autonomous airplane SHM nodes and the adjacent peripherals. In the first case, airflow EH devices formed by separate bodies will be reviewed. The first and most intuitive EH modality in this case is a rotary micro-turbine which converts mechanical rotation, via electromagnetic induction, into usable electrical energy [92]. The second EH modality utilizes airflow-induced fluttering and converts the resulting mechanical into electrical energy by using, in most cases, piezoelectric devices, although electromagnetic and electrostatic transducers can be employed in this frame as well [41,92,194,195]. The second EH principle that will be considered here are acoustic EH systems based on particular design arrangements on aircraft (cavities and similar), inducing sound wave effects [196,197].

### 5.1. Rotary Micro-Turbines

Although the rotary turbine principle can ensure a very stable power source, and it is commonly used in larger realisations in modern aircraft, i.e., as an emergency power source for the case of potential failures of the main system [198], when it comes to the miniaturization of the overall design so as to create practical EH devices for SHM power supply purposes, the efficiency of such devices, whose application in river flows, in the form of a propeller suitably coupled to a miniaturised DC generator, was recently successfully demonstrated [92], could be lower. In order to roughly estimate, then, the size of the key element (propeller) of such a system enabling the attainment of the required harvested power levels, simulations based on the simplified first-order mathematical formulation [198] are performed in the MATLAB^®^ environment, considering also that it is not possible to convert all available airflow energy into mechanical work [199]. The output power of the micro-turbine can thus be calculated as:

(8) $$P=\frac{1}{8}\cdot C_{p}\cdot \rho \cdot d^{2}\cdot \pi \cdot v^{3}$$

where $C_{p}$ is the power coefficient defining the fraction of wind power that is efficiently converted to the rotation of the propeller, limited by a theoretical upper limit of 0.593 (the so called Lanchester–Betz limit) [199], ρ is the air density, *d* the diameter of the propeller, and *v* is the airflow velocity.

**Figure 31:**
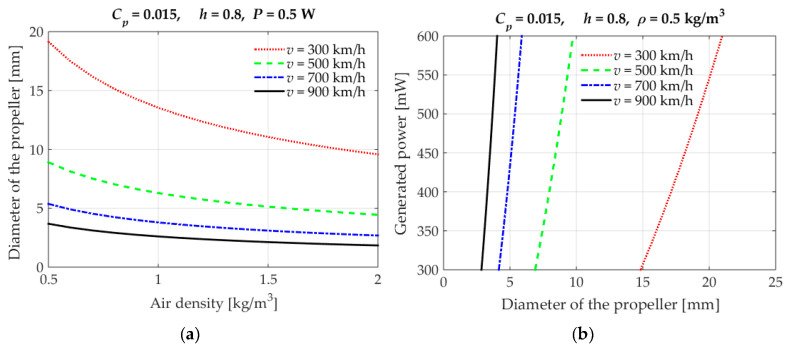
Diameter of the propeller vs. air density (**a**) and generated power vs. propeller diameter (**b**) for different aircraft velocities.

The electrical power that will be generated at the output of the micro-turbine can finally be calculated by multiplying the generated mechanical power of Equation (8) by the efficiency *η* of the electrical generator. Supposing, then, that the required output electrical power in the herein considered case is 0.5 W, and for aircraft velocities ranging from 300 to 900 km/h, in Figure 31a is depicted the resulting dependence of the propeller diameter on air density (i.e., flight altitude and temperature). Figure 31b shows the respective dependence of the generated power on a propeller’s diameter, again for different airflow velocities.

The performed first-order rough estimates allow, hence, establishing that very small propeller diameters suffice to achieve the power levels of a few hundred mW needed for the considered autonomous wave propagation SHM sensor nodes with the coupled data elaboration and wireless transmission modules. In fact, since power increases with the third power of airflow velocity (cf. Equation (8)), and given the relatively high flying velocities of the considered commercial airliners, suitable fixed or variable angle (active) physical airflow dampers (flaps, shutters) will have to be installed in front of the micro-turbine to obtain reasonably (few mm) sized EH devices of this type, so as to enable their easy handling and mounting, while minimizing the effect that the turbines, mounted in a suitable position, possibly on the lower part of the wings or the fuselage, would have on the aerodynamic performances of the airplanes themselves. A possible alternative could be to allow the rotation of the turbine only in lower-velocity regimes (i.e., during airplanes’ take-off or landing) and collect the thus generated energy in an appropriate storage element so that it can be used when needed (cf. in this regard Section 7 below dealing with power management electronics and respective storage elements). Additional thorough analyses are, in any case, needed to investigate all these (and other, e.g., problems related to protection from dust, ice and similar environmental effects) design aspects crucial to enable the evidently big potential of this EH technology in aircraft.

### 5.2. Air-Structure Interaction of Oscillating Bodies

A review of the current prospects and future trends for aerodynamic EH in aircraft applications, with considerations on the layout and advancement of EH devices based on aerodynamic instabilities, was recently performed [41]. The underlying principles rely generally on airflow (von Karman) vortices induced by obstacles (i.e., bluff bodies) or aerofoil sections that induce the fluttering or galloping of structures placed in such an environment (cf. in this regard also the treatise on nonlinear and bistable/multistable PEHs in the above Section 2.2.1). In this context, fluttering refers to self-feeding vibrations, where the aerodynamic forces on an object couple with its dynamical eigenmodes to produce rapid periodic motions. Galloping is, in turn, a velocity dependent, low frequency and large amplitude oscillatory aeroelastic instability of light and lightly damped slender structures, usually of non-circular cross sections. In any case, the used excited structures are, again, generally piezoelectric.

**Figure 32:**
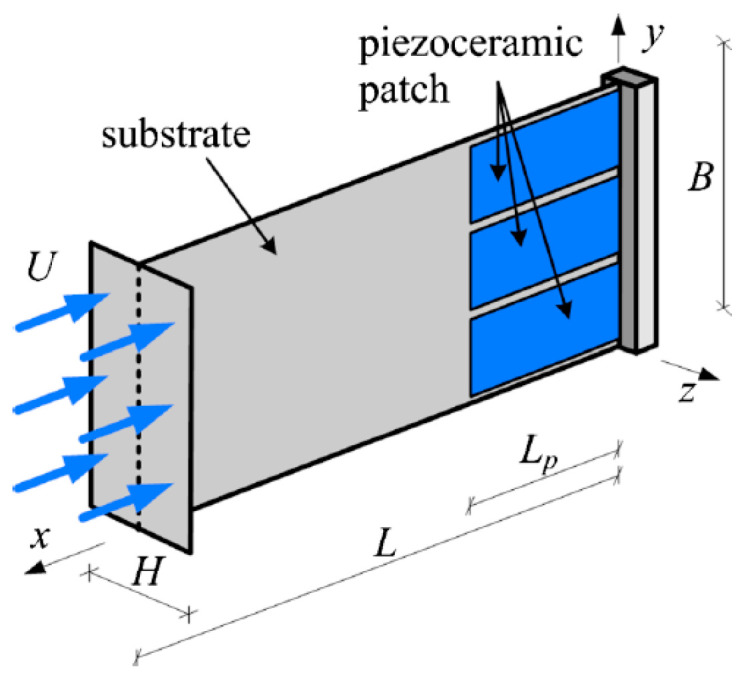
T-shaped airflow fluttering PEH [201].

A proof-of-concept experimental demonstration of the usage of ambient airflow, at an air speed of 26 m/s, to power aerodynamic control surfaces, via a small EH piezoelectric beam and an aeroelastic flap at the trailing edge of a wing section, was, hence, shown to allow producing average power levels of more than 40 mW [200]. Six flexible 28 × 14 × 0.3 mm^3^ piezoceramics patches, attached next to the fixture of both sides of a 100 × 60 × 30 mm^3^ T-shaped cantilever structure (Figure 32), subjected to a 4 m/s (ca. 14,5 km/h) airflow, inducing a 6 Hz aerostatic fluttering, generated then, on a resistive load of 4 MΩ, a peak power output of 4 mW (i.e., 0.567 mW/cm^3^ and 1.04 mW/g, respectively) [201]. A promising but quite complex design configuration, involving additional magnets, is that of a Y-shaped bistable EH device with piezoelectric patches aimed at low-speed (3 to 7 m/s) airflows that was experimentally shown to generate noteworthy voltage outputs [202].

**Figure 33:**
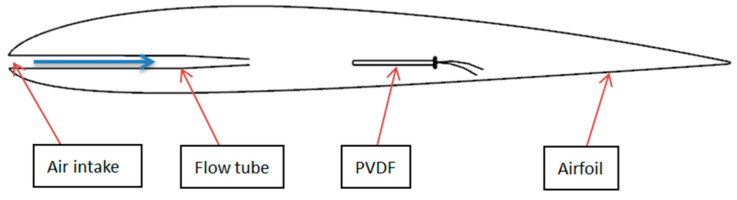
Scheme of jet-edge EH device in an aerofoil [194]-licensed under a Creative Commons Attribution (CC BY).

The design and characterization of a novel airflow EH device, based on jet-edge flow oscillations (Figure 33), proposed in [194], allows, in turn, attaining, via the oscillations of a polymeric (PVDF) piezoelectric structure (cf. above Section 2.3.2), in absolute terms only several tens of µW of maximal output power. The respective normalised power densities are, however, high, while the structure is rather simple.

**Figure 34:**
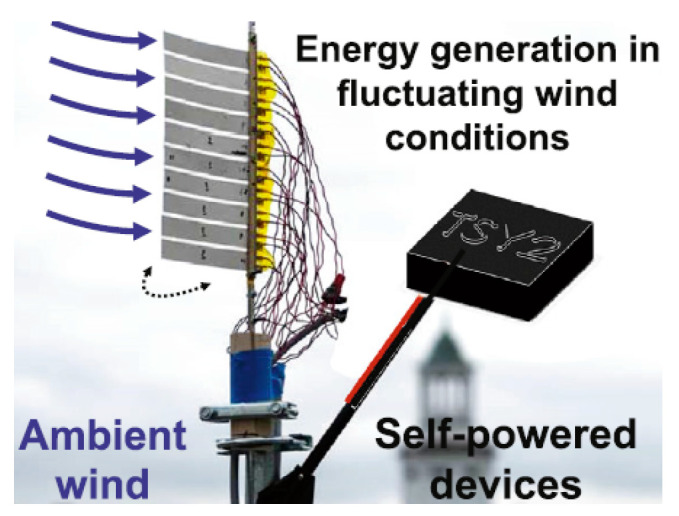
Principle of the PVDF EH generator based on an ‘inverted flag’ [195].

Several configurations that in the future could potentially enable the use of PVDF EH systems as structures on an airplane wing or the aircraft construction in general, were also investigated. A systematic experimental study of the influence of geometrical parameters on the behaviour and power output of a PVDF EH ‘inverted flag’ (Figure 34; In literature referred to as ‘piezoelectric eel’ [88]) allowed, then, to establish that, at an airflow velocity of 9 m/s, maximal power outputs of up to ~5 mW/cm^3^ can be obtained [195]. One of the ingenious design configurations used in this context is based on an air amplifier (i.e., an airflow directing nozzle in front of the EH device) aimed at increasing the flow of air and the resulting energy conversion efficiency of a PVDF EH device with an additional aerofoil baffle at its free end. With a constant airflow of 200 l/min, such a system provided on a 7 MΩ resistor a maximal power output of 13 mW [203].

**Figure 35:**
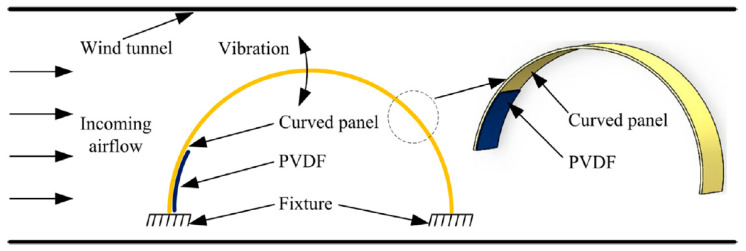
Scheme of the curved panel EH device concept [204].

Curved panels (already widely used in unmanned or micro aerial vehicles), with different portions of the semi-circular copper structure covered by segmented PVDF active piezoelectric layers, to be considered as part of aerofoil aircraft structures, have finally been used in a flutter-induced vibration EH device (Figure 35). Based on FE calculations and experimental tests performed in a wind tunnel, it was hence shown that, for an airflow of 25 m/s (90 km/h), a maximal harvested power of barely 0.42 μW (corresponding to a power density of 0.032 mW/cm^3^) can be obtained on a 10 MΩ resistance [204].

### 5.3. Acoustic Energy Harvesting

**Figure 36:**
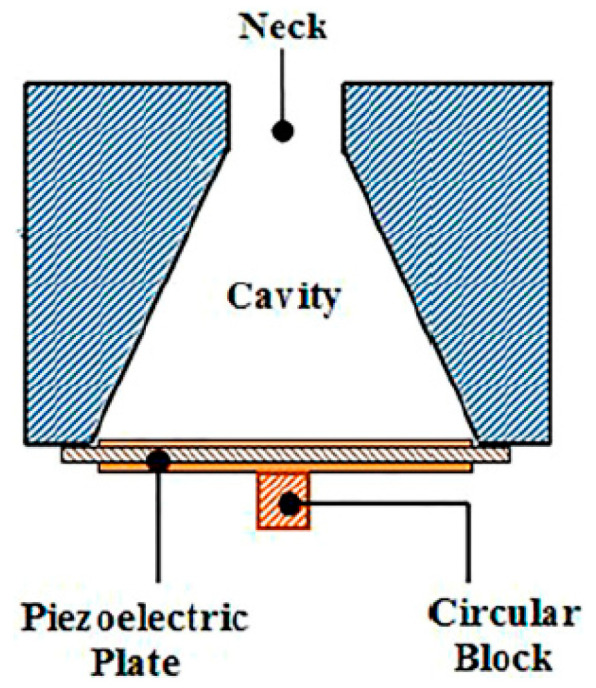
Helmholtz cavity-based AEHS with piezoelectric EH transduction [197].

Acoustic waves have been considered in aircraft structures mainly as viable means of performing SHM tasks [205]. Only very recently the concept of acoustic EH systems (AEHS) for wireless sensor networks, often based on Helmholtz cavities (producing resonance effects equivalent to those when blowing through the neck of an empty bottle) and piezoelectric elements (Figure 36–although the used oscillating structure could also be based on electromagnetic conversion), has been systematically reviewed [197]. However, even by using loud (pain threshold) ambient acoustic sources on the level of 130 dB (i.e., on the noise level of jet airplanes), AEHS of this type allow attaining limited absolute (with peak values on the order of 1 mW), and very limited specific power outputs (in the range of tens of μW/cm^3^) [196,197]. Such EH systems have thus been so far proposed only for an application in large surface noise barriers for high-speed railways [206].

**Figure 37:**
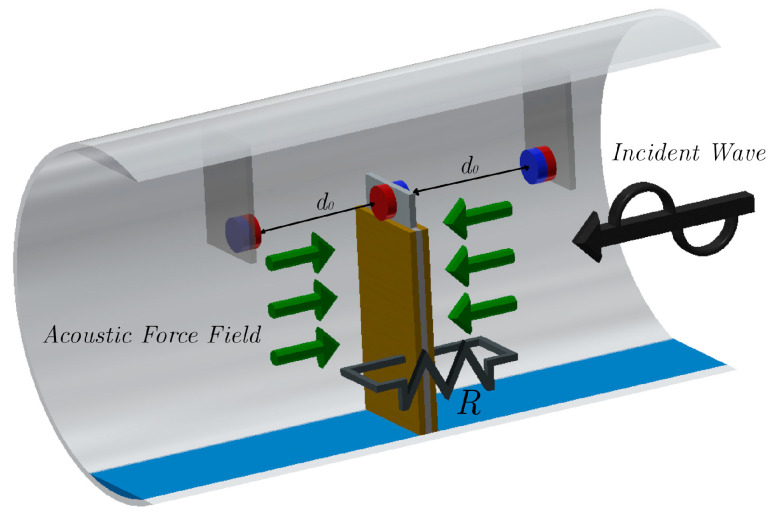
Quarter-wavelength tube AEHS concept with nonlinear restoring force arrangement.

A different, low-frequency broadband AEHS concept, is that of placing PEH devices in quarter-wavelength resonator tubes, i.e., those where, by adapting the length of the tube to the quarter-wavelength of the external sound wave, the incident sound pressure is amplified, i.e., a resonant standing wave is generated. The resulting maximal output powers are still, however, in the <1 mW range [197]. When a nonlinear restoring force is introduced into such a system via permanent magnets (Figure 37-cf. also the above Section 2.2.1), it is possible to tune the resonance of the bimorph PEH device to that of the cavity, despite its changes induced by temperature variations, thus optimising the performances of the device [207].

**Figure 38:**
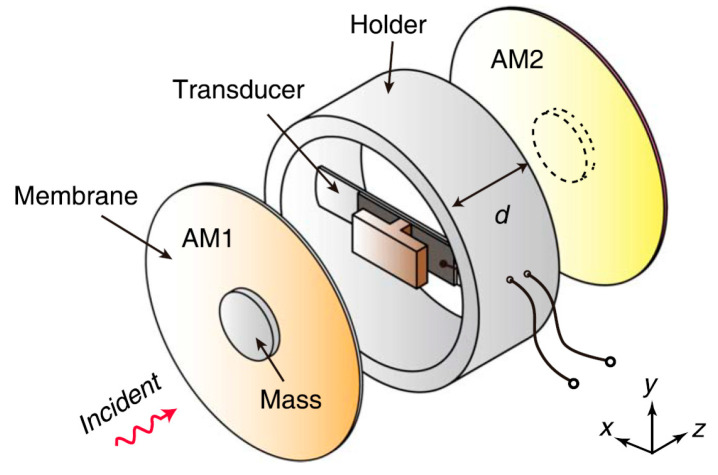
Scheme of an LAM-based AEH system [208].

A novel AEHS approach based on acoustic metamaterials, such as sonic crystals or layered acoustic metamaterials (LAM–Figure 38), allowing to attain sound focusing or amplification, thus contributing to the weight vs. EH efficiency potential that is critical, especially in airplanes, could provide further interesting development potentials [197], although with the reached maximal power outputs of only a few μW and power densities on the level of 0.5 μW/cm^3^ at best [208,209].

In any case, although conceptually interesting, AEHS are still generally at the proof-of-concept technology level and allow attaining very small power output densities, so that their actual applications for the herein considered autonomous SHM systems in airplanes are yet to be developed.

### 5.4. Summary of Findings on Airflow and Acoustic Energy Harvesting

In the above paragraphs it is shown that airflow EH devices, based on a practically unlimited energy well, show interesting potentials in providing the powers needed by the autonomous wave propagation SHM sensors nodes with the coupled data elaboration and wireless transmission modules. In fact, the fluttering/galloping EH devices, mostly based again on piezoelectric materials, allow attaining several mW/cm^3^ already at very modest airflow speeds of only a few tens of km/h, which makes them, despite the rather complex design configurations and issues related to aerodynamic resistance, an interesting prospective solution. If problems related to de-icing, the influence of dust particles or the high dynamics of the moving parts are thoroughly investigated, micro-turbines with propellers of only a few millimetres in diameter, protected by airflow dampers and properly positioned on the airplane, could certainly provide the need powers as well. Acoustic EH devices, although being a subject matter of rather intense recent investigations, enable, in turn, obtaining, even in the best cases, only very limited peak power densities (tens of μW/cm^3^), while their technology readiness levels are still low.

## 6. Radio Frequency Energy Harvesting and Wireless Energy Transmission Systems

**Figure 39:**
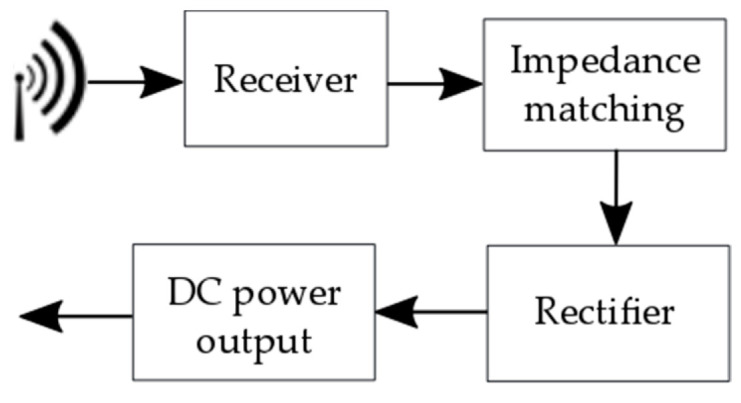
Scheme of an RF EH system.

Radio frequency (RF) is recently being considered as a prospective low-weight and widely available EH methodology to drive low-power wireless systems [189]. In fact, among the available ambient energy sources, RF signals could often be the most suitable source for wireless communication systems since vibrations, heat, light, and airflow are not consistently distributed in time and space [210]. RF EH devices convert the energy of human-generated (and thus dependant on the design of the whole considered construction) ultra-high frequency (generally in the range from 300 MHZ to 3 GHz) electromagnetic waves into a DC output voltage (Figure 39) to power not only low-power consumer electronics, including environmental sensing, but also signal processing and wireless communication components. In this regard it is perhaps more appropriate to designate this methodology as energy supply more than EH in the classical meaning of this term. RF systems provide, therefore, simultaneous wireless information (WIT) and wireless power transfer (WPT), i.e., they deliver wireless information and controllable energy in the same RF signal [211] even to places in, e.g., airplanes that are difficult to access with other considered EH systems [60]. The integration of WIT and WPT has attracted much interest in a wide range of commercial and military applications [212] and, in the herein considered application, could allow the delivery of energy via radio waves from the airplane cockpit or the passenger cabin (i.e., any position where a suitable emitting antenna could be placed) to the SHM sensor node and adjacent data elaboration components along the wings or other critical composite airplane structures, and the transmission of the respective data back to the cockpit.

The WPT itself can be based, in this frame, on a near-field or a far-field concept. The near-field concept utilizes either inductive or capacitive coupling, typically operating at 135 kHz or 13.56 MHz, with a range of up to 1 m for the inductive power transfer and up to 1 cm for the capacitive power transfer. The attenuation of the inductive power transfer, for a distance *d* between the emitter and the receiver, varies then with 1/*d*^3^, i.e., 60 dB per decade [213]. The near-field coupling of the transmitter and the receiver implies also that, to optimize the power transfer efficiency, both the possibly variable load and the coupling have to be properly considered. The far-field concept is, in turn, based on a specially designed RF rectifying antenna, referred to as rectenna, which is used to collect the energy from the distant sources of radiated electromagnetic fields. In this case, the attenuation is proportional to 1/*d*, i.e., it is 20 dB per decade. The RF EH technology is, thus, highly distance dependent so that, compared to other ambient energy sources, the available power density of the far-field RF EH technology is significantly lower, with the expected power densities in the 0.2 nW/cm^2^–1 µW/cm^2^ range [56], whereas that of the near-field technology could reach, in the best cases, tens of μW/cm^2^ [189], which, compared to the above considered EH methodologies, is still ca. two orders of magnitude lower.

**Figure 40:**
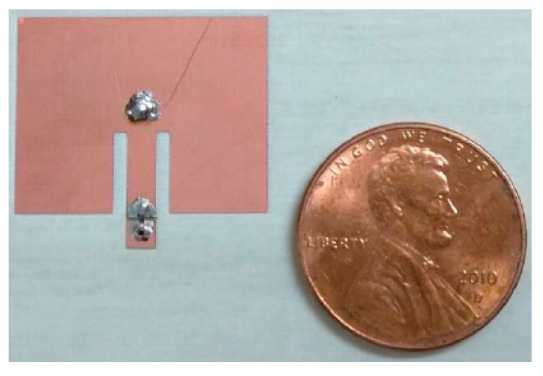
Low-power/low duty cycle rectenna RF EH device powered by a nearby aircraft altimeter radar [215].

The RF EH is, therefore, an option only for very low-power sensor systems. A study of the WPT as an EH source for sensors in the fuel tank of an airplane has recently been carried out, demonstrating its feasibility [214]. In another use of the RF concept, a rectenna-based EH device (Figure 40) for low-power/low duty cycle aircraft sensors is placed on the aircraft skin on the lower part of the fuselage, collecting successfully the energy of a radar altimeter operating at 4.3 GHz. For the rectenna positioned at 30 cm from the altimeter with a transmission power of 0.5 W, the EH power density could in this case reach 2.2 µW/cm^2^ [215].

**Figure 41:**
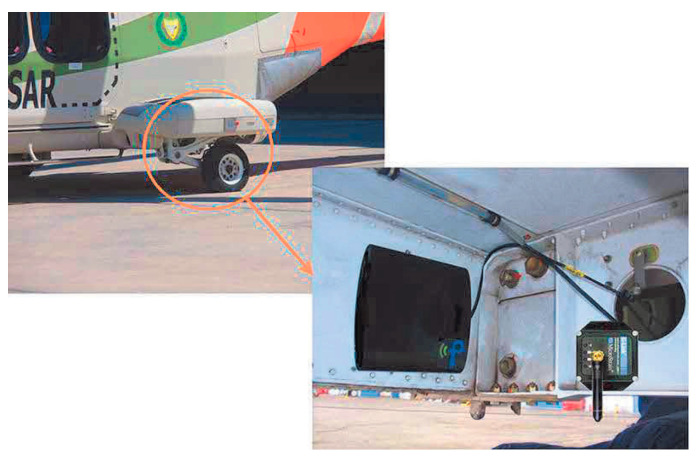
RF collector and wireless sensor in the landing gear unit of an AW139 helicopter [216].

An experimental approach to RF energy harvesting in the far-field was, in turn, demonstrated by using a commercial Powercast Lifetime Power P2110 rectenna operated at 900 MHz mounted on an AgustaWestland AW139 helicopter landing gear compartment (Figure 41), allowing a DC power output of 70.42 µW to be transmitted from a 76 m distant GSM base-station to an accelerometer sensor node in the helicopter [216].

**Figure 42:**
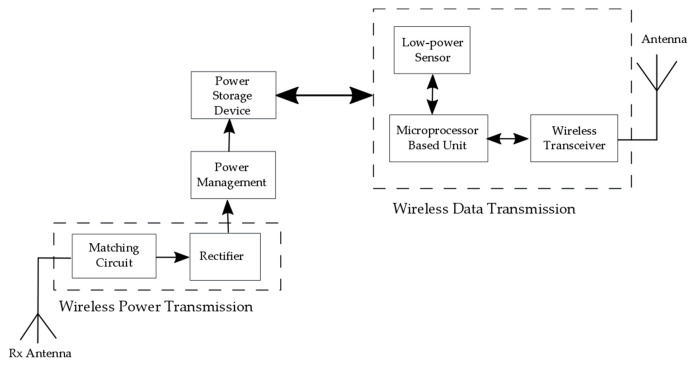
Block diagram of an RF-powered wireless sensor node with the data transmission system.

The potential of supplying power for SHM aircraft sensors via a microwave (915 MHz) RF signal has been experimentally explored in [217], where 200 µW were successfully delivered over 1 m to two linear-pattern resistive strain gauge sensors, also assuring the transmission of the respective data back to the base station. The microwave energy was captured in this case by two kinds of receiving antennas (dipole and patch), evaluated by impedance matching and peak received powers, transformed into DC power by a rectifying circuit, and stored in a supercapacitor to provide the energy required by the wireless sensor nodes (cf. Section 7 below) to be potentially located in structures such as wing skin, airplane engine, stiffener, or similar (Figure 42).

The above examples therefore clearly show the potential of RF wireless energy and data transmission systems, especially for sensors placed in locations that are difficult to access with other considered EH devices, although they are not an immediate solution for the wave propagation SHM sensors nodes with the data elaboration and transmission modules described in the introduction of this work, which still require far higher power levels. As pointed out, the other components of the ODIN EU COST Action are actively working on the optimization of the placement of the sensors, the minimization of the respective power consumption, and the optimization of the signal processing protocols, as well as data transmission rates, which could all contribute to decrease the needed power levels, thus possibly enabling the usage of some of the currently not usable EH modalities.

In any case, this concludes the thorough review of the currently available EH technologies but, to incorporate them in the foreseen autonomous in-process SHM systems in airplanes, the respective power management and energy storage elements have to be carefully considered as well. The review of the current state-of-the-art of the latter will thus be carried out in the following section.

## 7. Power Management Electronics and Energy Storage Elements

**Figure 43:**
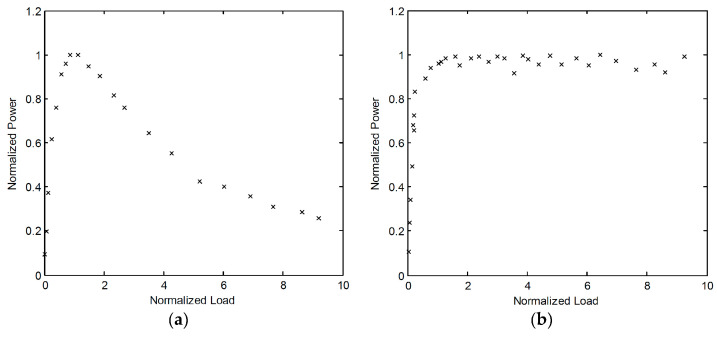
Normalized power obtained in experimental results for a PEH with different attached loads without (**a**) and with (**b**) an adaptive power management circuitry [218].

As evidence above, depending on the used EH modalities and the environmental conditions, the produced output voltages can vary considerably in amplitude (for both AC (kinetic, RF and most of the airflow EH devices) and DC (TEG, PV and the micro-turbine EH modalities) sources) and frequency (for AC sources). In order to feed the energy to the foreseen electrical load (in the case considered in this work the SHM sensor node with its peripheral components), an appropriate management electronics is, therefore, necessary. Power management devices coupled to EH systems have, thus, several functions. Firstly, they enable the adjustment or matching of the voltage from the EH transducer to that of the load which is being powered–typically ranging from 2 to 5 V with currents of up to 100 mA [32]. A further task is the regulation of the supply voltage, i.e., keeping it as much as possible constant and independent of the source’s or load’s fluctuations. Last but not least, the power management electronics assures an efficient energy delivery from the EH device, i.e., the generator, to the SHM system (the load), as well as the storage of the surplus harvested energy on an appropriate storage device and its management [2,66]. The key solution in this regard is, hence, tailoring as much is possible the power management electronics towards the EH device and its operation while aiming at obtaining the maximum power transfer (i.e., at minimizing the energy consumption on the electronics itself). Efficient adaptive electronics can, for example, be designed with the capability of an effective power transfer with a wide range of attached loads (Figure 43) [218].

Power management devices typically encompass therefore the following main components:

- A highly efficient DC-to-DC converter used to adjust the voltage amplitude to the needed level as well as for impedance matching (i.e., adapting the input impedance to the maximum power point of the harvester) [66]. DC-to-DC converters also often have the function of boost converters (multipliers).
- The power management circuity that often integrates a low-loss (i.e., with a voltage drop of barely 0.7 V) full-wave bridge rectifier for the conversion of AC to DC voltage (for EH devices producing AC output voltage), the maximum power point tracking (MPPT) unit (setting the required input voltage value, thus allowing to extract the maximum available energy that an EH generator can produce), and the ’cold start’ circuitry (used, rarely, to initialise the operation of the EH device) [2,219,220]. Several commercially available solutions of this type are available as off-the-shelf products [32]. Examples of circuitry that can be used in specific applications are also state-of-charge (SoC) monitoring devices that are used to control the load based on the available energy so that it is operated only when the harvester generates appropriate energy levels.
- An energy storage device in the form of a polarized capacitor, a super-capacitor, a battery or a hybrid solution [219].

**Figure 44:**
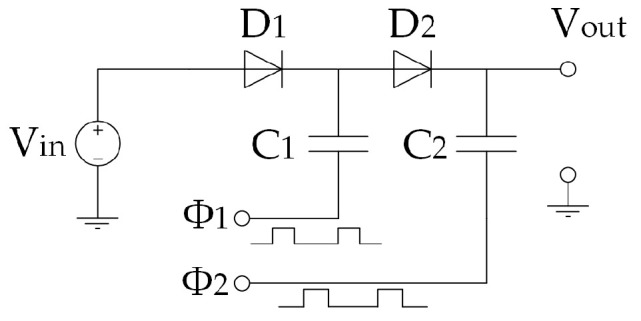
A Dickson charge pump.

The design of DC-to-DC boost converters with very low (below few hundred mV) start-up voltages is still quite challenging. There are then two types of DC-to-DC boost converters commonly used with EH devices having low-voltage DC outputs: a charge pump or an inductor-based boost converter [221]. The charge pump (or switched capacitor) converter utilizes diodes (or diode-connected transistors) and capacitors to transfer the charges through the charging and discharging phases. In Figure 44 is hence depicted the Dickson charge pump voltage doubler topology [222]. The advantage of the charge pump is that it can provide an integrated solution in a small volume, since the capacitors can be fully integrated in an on-chip configuration. The efficiency of this boost converter type is, however, limited due to the inherent energy losses when a charge is transferred from one capacitor to another, which are present even when all the components of the circuit are ideal [223].

The inductor-based boost converter in turn utilizes switches and an inductor to store the energy and transfer it to the load, and is generally used in applications with wide input voltage ranges. Theoretically, this configuration can achieve a 100 % power efficiency and it provides a high boost ratio. It is, therefore, widely used e.g., in TEG-based EH applications [224]. The factual efficiency of the inductor-based boost converter depends on the quality factor of the inductor, the control power losses, and the employed technology [225]. The efficiency is limited also for very low input voltages, when the converter needs additional cold-start circuitry [219,220]. What is more, the inductor cannot in this case be integrated in an on-chip configuration, since its design requires an inductor’s value in the μH range. There is, therefore, a trade-off between the respective integration potential and power efficiency.

Two types of inductor-based boost converter can then be distinguished, based also on the required duty cycle of the foreseen load (i.e., the respective times of operation of the load and the times spent in the switch-off state): the asynchronous and the synchronous boost converters.

**Figure 45:**
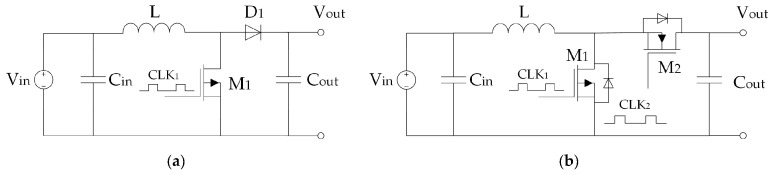
The asynchronous (**a**) and the synchronous (**b**) inductor-based boost converter.

The asynchronous boost converter is shown in Figure 45a. The respective output voltage depends on the duty cycle D of the switch M_1_, i.e., of *clk*_1_, so that [225]:

(9) $$V_{out}=\frac{V_{in}}{1-D}$$

The advantage of the asynchronous boost converter is that only one clock signal is required, which makes the design easy to implement. The diode degrades, however, the resulting power efficiency due to its internal resistance and the resulting voltage drop.

To improve the power efficiency and reduce the voltage drop across the diode, in the synchronous inductor-based boost converter the diode is replaced by a PMOS transistor (Figure 45b). The output voltage on this type of boost converter depends on the on-times of the NMOS and PMOS transistors, i.e., *T_nmos-on_* and *T_pmos-on_*, respectively [225]:

(10) $$V_{out}=\left(1+\frac{T_{nmos-on}}{T_{pmos-on}}\right)\cdot V_{in}$$

Due to their very low efficiencies, transformer-based and switched capacitor DC-to-DC boost converters are generally not used in EH-based applications.

As evidenced above, by exploiting ambient dynamical (oscillatory) mechanical energy, PEHs and similar EH devices generate an AC voltage that cannot be used directly to power the load, i.e., the autonomous SHM sensor nodes with adjacent peripherals. A suitable power management circuitry is thus required in this case to rectify, store, and regulate the energy produced by such EH sources. The goal of EH electronic interfaces is, hence, to efficiently extract the energy from the electromechanical harvesting structures, which implies the need to minimize the dissipation of energy on the power management unit itself. The latter must, therefore, be designed and optimized considering stringent low-power requirements. Different rectifier configurations aimed at achieving such a goal have been proposed and recently thoroughly reviewed in [226]. The most important findings of this review are shortly summarised below.

**Figure 46:**
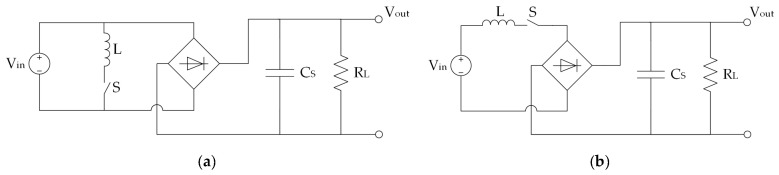
The S-SSHI (**a**) and the P-SSHI (**b**) rectifiers.

Rectification is usually done by employing passive interface circuits such as a full bridge rectifier, a voltage doubler or a negative voltage converter. In the case of passive interface circuits, the voltage drop on the diodes has to be taken into account. A MOSFET bridge solution can, in turn, be used to achieve an active rectification. The series synchronized switch harvesting on inductor (S-SSHI) and parallel synchronized switch harvesting on inductor (P-SSHI) were the first two resonant rectifiers proposed in this frame. A switch S (in practice represented by a semiconductor device, i.e., a transistor) and an inductor L can, thus, be connected here to the PEH element in series (Figure 46a), or in parallel (Figure 46b), respectively [226].

**Figure 47:**
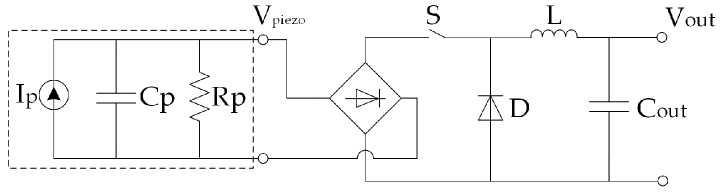
The SSDCI rectifier circuit.

Voltage inversion causes an electrical attenuation that opposes the vibrations-induced mechanical dynamical strain of the piezoelectric material. This effect, referred to as synchronized switch damping (SSD), significantly affects the overall conversion efficiency, particularly in strongly coupled PEH transducers. Due to the direct connection of the switched components to the PEH transducer, the SSD effect is indeed one of the main problems of both the S-SSHI and the P-SSHI rectifiers. In the synchronized switching and discharging to a storage capacitor through an inductor (SSDCI) rectifier, the diode bridge rectifier is, in turn, directly connected to the PEH (Figure 47), thus preventing the current flow that causes the evidenced electrical attenuation [226].

**Figure 48:**
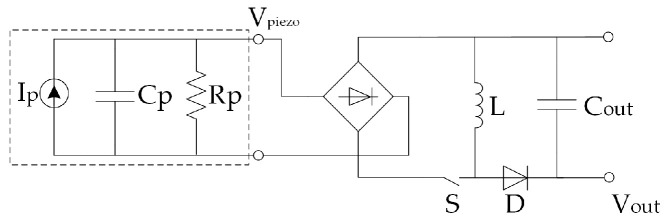
The SECE rectifier circuit.

The synchronous electric charge extraction (SECE) technique is, on the other hand, a rectifier configuration similar to that of a buck–boost power converter, but with a different switch control (Figure 48). The SECE technique is intrinsically self-adaptive to the environment even in the case of random vibrations, and it is less sensitive to changes in the load, which makes it particularly attractive in the applications considered in this work [226].

**Figure 49:**
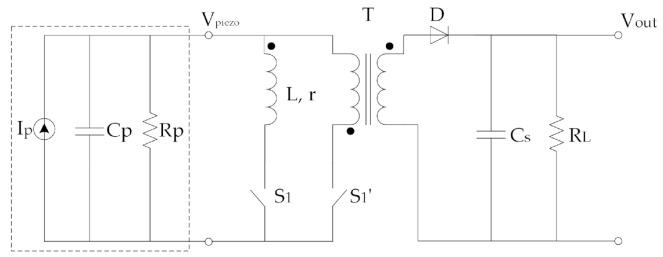
The MR-SSHI rectifier circuit.

Using the SECE technique, the regulation of the trade-off among power extraction and the damping effect is, however, still unattainable. To overcome this limitation, the phase shift synchronous electric charge extraction (PS-SECE) rectifier was proposed. In the synchronized switch harvesting on inductor magnetic rectifier (MR-SSHI) the switching inductor is replaced by a step-up transformer that electrically decouples the storage capacitor from the PEH (Figure 49), and, since its coupling factor is chosen to be >1, it reduces the diode voltage drop reflected back to the PEH, increasing thus the value of the EH device’s voltage. The MR-SSHI rectifier configuration is, therefore, particularly suited to extract the energy in the case of very low input power levels [226].

**Figure 50:**
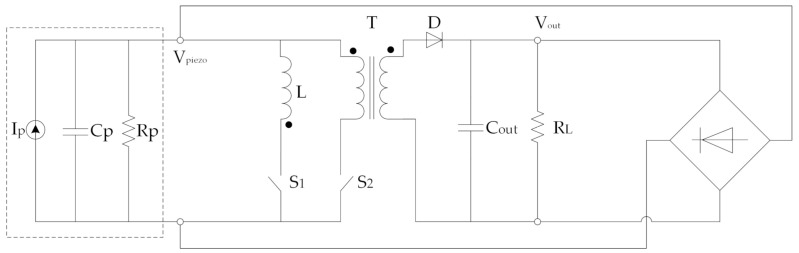
The Hybrid SSHI rectifier circuit.

The hybrid SSHI rectifier (Figure 50) is a configuration derived combining the MR-SSHI rectifier, mostly effective for high load values, and the P-SSHI rectifier, mainly effective for middle load values. In fact, by coalescing these two approaches it is possible to extract energy in both the inversion and the conduction phases. The hybrid SSHI circuit does not improve conversion efficiency, but, due to the doubled harvesting rate, it allows widening the attained load bandwidth [226].

**Figure 51:**
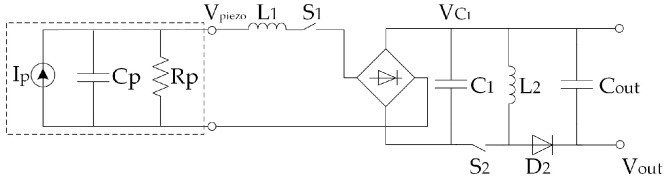
The circuit of the DSSH, ESSH and ASSH rectifiers.

The double synchronized switch harvesting (DSSH) is, finally, a rectifier derived from the S-SSHI and the SECE ones where, analogously to the SECE configuration, the harvested power is almost independent on the connected load. The advantage of the DSSH with respect to the SECE configuration is that, by finely tuning the value of the ratio of the PEH capacitance *C_p_* to that of the intermediate capacitor *C_int_*, the trade-off between the damping effects and harvested energy can be controlled. The same circuit topology can then be used to implement also the enhanced synchronized switch harvesting (ESSH) rectifier where, with respect to the DSSH one, *C_int_* always stores a small amount of energy. The ESSH rectifier has, thus, a lower sensitivity to the capacitance ratio *C_int_*/*C_p_* mismatch. The adaptive synchronized switch harvesting (ASSH) is an optimization of the ESSH rectifier designed particularly for multi-mode vibrations of EH device. DSSH, ESSH, and ASSH rectifiers then all have the same circuit topology (Figure 51), but they differ in the control method of the switches *S*_1_ and *S*_2_ [226].

All of the above SSHI (synchronized switch harvesting on inductor) approaches (S-SSHI, P-SSHI, MR-SSHI, and Hybrid SSHI) are strongly dependent on the connected load. In real circuit implementations, SSHI rectifier types usually require, thus, an additional DC-to-DC conversion stage to keep the rectified voltage constant. With respect to the SSHI and energy injection techniques, rectifiers of the SECE, DSSH, and ESSH types have, in turn, a more constant behaviour [226]. In any case, future research foci should primarily be on the minimum operating voltage criterion (the performances achieved in implementations focusing on this aspect are still limited), as well as on increasing the energy efficiency on each stage of the power converter.

The energy attained from EH devices, suitably adjusted/rectified via the above illustrated electronic elements, is finally fed to an energy storage device such as a capacitor or a rechargeable battery so that it can be used when needed, thus overcoming the often-existing dynamical power mismatch between the generator and the load [219]. Capacitors, in a broad range of types (Li-ion, ultra, double-layer (supercapacitor), electrostatic, electrolytic, etc.), are then mostly used as a peak power source for applications where the energy is utilized during (or briefly after) the operating stage of the EH device. DC-to-DC converters are used in this case to deliver a higher-level voltage to the capacitor, while additional electronics can be required for the stabilization of the output voltage. Energy storage based on capacitors is generally regarded as maintenance free, since capacitors can withstand lifetimes of millions of cycles and service lives in the range of 10–15 years of operation [2].

When rechargeable batteries are used instead, the energy is supplied to the load (the SHM sensor node) even when the EH device is not operating. Batteries (especially the commonly used lithium ones, requiring the control of both voltage and current) require power management electronics that comprise elements used to assure their charging control and the protection from under- or overvoltage levels. The resulting power management electronics can be assembled from commercially available components (which is generally the case for high-power applications–at the level of tens of W and higher), or, for lower power levels, it can be purchased as a specific ‘surface-mount technology device’ (SMD), with all the electronics elements integrated onto the surface of a printed circuit board (PCB). Batteries, which can generally be in the form of Li-ion, Li-ion-polymer, lead-acid, Ni-MH or Ni-Cd ones, require maintenance as well [2]. The batteries considered appropriate for energy storage in EH-based applications have a capacity range typically from 1 to 4 Ah (i.e., approximately from 3.5 to 14 Wh) [1]. If a constant power of 100 mW would, thus, be required in a specific SHM application on an aircraft, a fully charged battery with a 10 Wh capacity would, therefore, theoretically last for four days. This confirms once more the evidenced importance of carefully considering the optimal duty cycle of operation of the SHM sensor nodes and the respective data elaboration and wireless transmission modules.

It has to be noted in this context also that the temperature range of operation is a very important aspect in choosing the appropriate storage device to be used in aeronautic applications as well. In fact, although Li-ion rechargeable batteries that are characterised by a high energy density and a low self-discharge rate, are commonly used, their operational temperature range is limited from ca. −20 °C to ca. + 60 °C. Besides a lower energy density and high self-discharge rate, Ni-MH batteries have an even more limited temperature range (0 to + 60 °C). Compared to batteries, supercapacitors are characterised by a smaller energy density and higher self-discharge rates, but their lower operational temperature limit is down to −40 °C, so that they might be a more appropriate solution for aircraft applications [1].

One of the disadvantages of commercially available supercapacitors is, however, their voltage rating, which, due to leakage currents and resulting discharging characteristics, is lower for higher capacities (they are usually rated at 2.5 V). This implies that several supercapacitors have to be connected in series to obtain suitable voltage levels for the applications considered in this work [227]. A possible solution for energy storage that is sometimes considered is, thus, a hybrid solution, where a battery and a supercapacitor are combined, usually in a parallel connection. Since supercapacitors are characterized by low energy density, while batteries suffer from low power density, their connection, with respect to a single element, leads to a system with higher energy and power densities. An important advantage of hybrid systems is also that the battery does not power the load directly, so there are no sudden battery voltage drops associated with short-term higher current needs [2].

The most important elements and the respective main characteristics of the power management electronics and the corresponding storage devices, used in EH-based devices and systems, have, thus, also been reviewed. The above treatise shows, hence, that in this context the respective critical elements, are the DC-to-DC boost converters, with the advantages of inductor-based synchronous boost converters clearly evidenced, and low-loss AC-to-DC rectifiers, where it is shown that the SECE and DSSH-related rectifier configurations could be particularly suitable for the applications in SHM airplane systems considered in this work. Concerning the energy storage elements, it is, in turn, shown that capacitors, to be used as peak power sources, are a maintenance free long-life solution that can be used at low temperatures-a very important aspect in applications in airplanes, but are characterised by limited energy densities. Batteries, can, in turn, be used even when the EH device is not operating, but they require additional electronics, need maintenance, can be used in limited temperature ranges and are characterised by lower power densities. Hybrid solutions are thus sometimes considered as an appropriate solution, while developments in the ongoing research on high capacity and fast charging batteries are also to be monitored.

The readers have therefore now been provided with a complete overview of the most important features of all the components considered in this work. The resulting conclusions can hence be drawn.

## 8. Conclusions and Outlook

A detailed review of the prospective EH technologies to be used for powering integrated SHM systems in airplanes (especially their composite components), thus enabling a weight reduction and an increased fuel consumption efficiency, while reducing the MRO (maintenance, repair, overhaul) costs of aircraft via predictive maintenance, is given in this work as part of the activities carried on in the framework of the EU COST Action CA18203 “Optimising Design for Inspection” (ODIN). A description of the hence used SHM technologies, with an estimate of the needed power levels for a wave propagation autonomous in-process SHM sensor node with the coupled data elaboration and wireless data transfer modules, is thus given first, allowing to determine that in this case powers on the level of several hundred mW would be needed. EH devices investigated in the most up-to-date literature that could provide viable means for satisfying these requirements in the given operational conditions are therefore thoroughly reviewed next. The broad study of all the main potential EH modalities to be used for powering the autonomous SHM sensor nodes in airplanes with their peripherals, allowed hence establishing clear guidelines for the selection of the most appropriate ones in specific applications, evidencing at the same time their inherent advantages but also some limitations. The large number of presented state-of-the-art design configurations also provides a stimulus and inspiration for further innovations in this field.

It is hence shown that, due to abundance of dynamical excitations on airplanes, kinetic EH devices, usually based on piezoelectric or electromagnetic transduction, are a very attractive and broadly investigated solution, provided that their inherent advantages and drawbacks, summarised in the above Section 2.4., are duly considered and the devices are mounted in the vicinity (enclosure or mounting fixture) of the airplane engine, i.e., in locations with stable and predictable dynamical excitation conditions. Magnetostrictive, electrostatic, and TENG devices have, in turn, their inherent limitations and their technology readiness is not yet at the level required by the aeronautic industry.

Due to the presence of significant thermal gradients on-board of an aircraft, especially along the aeroengines, TEG devices, a rather mature technology proven on test flights, are shown to be also an attractive EH solution in airplanes, allowing potentially high power densities to be achieved (cf. Section 3.3.). Due to low conversion efficiencies, their usage in areas where medium or low temperature differentials are present could be interesting only when coupled to low-power electronics, provided further research work to reach higher TRLs for aircraft integration is carried on.

PV technology, with the large airplane surfaces where it can be used, has been proven as efficient means to power whole specially designed airplanes, while its usage for specific energy needs in commercial airplanes is matter of recent investigations only. In fact, although the efficiency of eventual PV EH applications in aircraft is highly dependent on the used PV cells and on uncontrollable environmental influences during flight, the very high attainable maximal power densities make the potentials of PV EH devices very interesting for much wider systematic investigations.

Airflow EH devices, based on a practically unlimited energy well, show interesting potentials. With a proper consideration of aerodynamic resistance issues and other design constrains related to their potential usage in factual aeronautical applications, oscillating bodies and, in perspective, micro-turbines, could thus possibly offer, promising possibilities, while acoustic devices allow attaining only very limited specific powers and their technology readiness is low.

The RF devices offer, finally, a means to deliver wirelessly the energy to SHM sensor nodes in locations that are difficult to access, but also assure the feedback on their operation. They allow, however, obtaining power densities at the level of only tens of μW/cm^3^, i.e., far lower than the other considered EH methodologies.

Provided, then, that the evident potentials of the vibration PEH and electromagnetic resonator devices, as well as of the high temperature gradient TEGs, are proven in the specific applications considered in this work, while the technology readiness of PV and some of the airflow (micro-turbine and, perhaps, fluttering/galloping devices) is improved, and taking into account the temporally changing ranges of optimal operation of these technologies, most probably the best solution could be the combined use of several of these EH devices so as to provide reliably and with sufficient safety margins the needed power levels of hundreds of mW even in the worst foreseeable operating conditions. However, this makes the task of selecting the most appropriate power management electronics configurations and the choice of the respective energy storage elements even more critical than what is the case when only a single EH device and/or EH technology is considered.

The review of the power management architectures provided in Section 8 shows hence the importance and relevance of inductor-based synchronous boost converters and of the synchronous electric charge extraction and the double synchronized switch harvesting AC-to-DC rectifiers in the herein considered application field. Capacitors are, then, to be used as peak power sources in low temperature conditions, batteries can be used when the EH device is not operating, while hybrid solutions, offering, concurrently, high energy and high power densities, are increasingly considered as a viable integrated solution.

The guidelines for the choice of the power management electronics and of the energy storage elements, when coupled to the provided guidelines on the EH devices themselves, as well as to the results of the active work of the other components of the ODIN EU COST Action on the optimization of the placement of the sensors, the minimization of the respective power consumption, and the optimization of the signal processing protocols as well as data transmission rates, create in perspective the preconditions for the development of a new class of autonomous sensor nodes for in-process non-destructive SHM of composite airplane components. The very broad list of the most recent bibliographical references provided at the end of the paper provides the perspective readers, in any case, the possibility to further deepen their understanding of all the aspects of the topics treated in this work.

## Figures and Tables

**Table 1:** Summary of used metrics for EH devices.

Reference	Metric	Expression
	Power density (specific power) (PD)	$PD=\frac{P}{V}$
[97]	Normalized Power Density (NPD)	$NPD=\frac{P}{A_{0}{}^{2}V}$
[140]	Figure of Merit (FoM)	$FoM=\frac{P}{A_{0}{}^{2}V}\cdot \Delta f$
[141]	Figure of Merit (FoM)	$FoM=\frac{P}{A_{0}{}^{2}V_{\Sigma }}\cdot \frac{1}{Q}$
[142]	Normalized Power Integral Density (NPID)	$NPID=\frac{P_{f}}{A_{0}{}^{2}V}$
Parameters: $A_{0}$ magnitude of harmonic excitation acceleration P useful power output $P_{f}$ power integral Q quality factor V reported total volume of the harvester $V_{\Sigma }$ volume of the harvester including the displacement Δf frequency bandwidth		
